# Post-Transcriptional Regulation of KLF4 by High-Risk Human Papillomaviruses Is Necessary for the Differentiation-Dependent Viral Life Cycle

**DOI:** 10.1371/journal.ppat.1005747

**Published:** 2016-07-07

**Authors:** Vignesh Kumar Gunasekharan, Yan Li, Jorge Andrade, Laimonis A. Laimins

**Affiliations:** 1 Department of Microbiology-Immunology, Feinberg School of Medicine, Northwestern University, Chicago, Illinois, United States of America; 2 Center for Research Informatics, The University of Chicago, Chicago, Illinois, United States of America; Penn State University School of Medicine, UNITED STATES

## Abstract

Human papillomaviruses (HPVs) are epithelial tropic viruses that link their productive life cycles to the differentiation of infected host keratinocytes. A subset of the over 200 HPV types, referred to as high-risk, are the causative agents of most anogenital malignancies. HPVs infect cells in the basal layer, but restrict viral genome amplification, late gene expression, and capsid assembly to highly differentiated cells that are active in the cell cycle. In this study, we demonstrate that HPV proteins regulate the expression and activities of a critical cellular transcription factor, KLF4, through post-transcriptional and post-translational mechanisms. Our studies show that KLF4 regulates differentiation as well as cell cycle progression, and binds to sequences in the upstream regulatory region (URR) to regulate viral transcription in cooperation with Blimp1. KLF4 levels are increased in HPV-positive cells through a post-transcriptional mechanism involving E7-mediated suppression of cellular miR-145, as well as at the post-translational level by E6–directed inhibition of its sumoylation and phosphorylation. The alterations in KLF4 levels and functions results in activation and suppression of a subset of KLF4 target genes, including *TCHHL1*, *VIM*, *ACTN1*, and *POT1*, that is distinct from that seen in normal keratinocytes. Knockdown of KLF4 with shRNAs in cells that maintain HPV episomes blocked genome amplification and abolished late gene expression upon differentiation. While KLF4 is indispensable for the proliferation and differentiation of normal keratinocytes, it is necessary only for differentiation-associated functions of HPV-positive keratinocytes. Increases in KLF4 levels alone do not appear to be sufficient to explain the effects on proliferation and differentiation of HPV-positive cells indicating that additional modifications are important. KLF4 has also been shown to be a critical regulator of lytic Epstein Barr virus (EBV) replication underscoring the importance of this cellular transcription factor in the life cycles of multiple human cancer viruses.

## Author Summary

Viruses that induce persistent infections often alter the expression and activities of cellular transcription factors to regulate their productive life cycles. Human papillomaviruses (HPVs) are epithelial tropic viruses that link their productive life cycles to the differentiation of infected host keratinocytes. Our studies show that KLF-4, originally characterized as a pluripotency factor, binds HPV-31 promoters activating viral transcription as well as modulates host cell differentiation and cell cycle progression. KLF4 levels and activity are enhanced in HPV-positive cells by E6 and E7 mediated post-transcriptional and post-translational mechanisms resulting in altered target gene expression and biological functions from that seen in normal keratinocytes. Importantly, silencing KLF4 hinders viral genome amplification and late gene expression. Along with its recently identified role in Epstein Barr Virus reactivation during differentiation, our studies demonstrate the importance of KLF4 in the life cycles of multiple human cancer viruses.

## Introduction

The life cycle of human papillomaviruses is dependent upon host cell replication, differentiation and cellular gene expression [1,2]. HPVs infect stratified squamous epithelia through small wounds that expose basal cells to entry. Upon entry, viral genomes are maintained as low copy nuclear episomes and replicate in synchrony with cellular chromosomes [2,3]. Following replication of infected basal cells, HPV DNAs are partitioned equally to the resultant two daughter cells. While one daughter cell remains in the basal layer, the other leaves the basal layer and begins to differentiate leading to productive viral replication, late gene expression, and virion assembly in suprabasal layers [1,2,4,5]. These processes are regulated by the concerted action of both viral and cellular transcription factors. These factors act either directly by binding to viral sequences in the early or late promoter regions or indirectly by modulating expression of host genes that provide critical functions for the differentiation-dependent HPV life cycle [2,3,6–10].

In undifferentiated cells, the early viral promoter (p97 in HPV 31 and 16) initiates transcription upstream of the E6 open reading frame (ORF) and directs expression of the E6 and E7 oncoproteins as well as the E1 and E2 replication factors [11–13]. E2 also acts as a repressor that auto regulates its own expression from the early promoter as part of a copy control mechanism [14–16]. Upon differentiation, the majority of viral transcription shifts to the late promoter located in the middle of the E7 ORF that directs high-level expression of E1, E2, E1^E4, and E5 along with the late capsid proteins, L1 and L2 [11,12,17,18]. While many cellular factors regulating early viral expression in undifferentiated cells, such as Ap-1, TEF-1, Sp-1, have been identified, the mechanisms and proteins that regulate late viral functions are still largely uncharacterized [17,19–24].

In addition to cellular transcription factors, microRNAs (miRNAs) also regulate viral and cellular gene expression. While HPVs do not encode their own miRNAs, they modulate the expression of a variety of cellular miRNAs [25–28]. One HPV regulated cellular miRNA is miR-145 which has been shown to be a negative regulator of the HPV31 life cycle [26]. Suppression of miR-145 expression in suprabasal epithelial cells by HPV proteins is necessary for differentiation-dependent viral DNA amplification and late gene expression. miRNAs have multiple targets in cells and miR-145 is one of the only miRNAs that has target sequences in the E1 and E2 open reading frames of HPV-31 with similar elements present in most HPV types. miR-145 also regulates the expression of several host genes including KLF4 [26], which is a major downstream effector of the p63 pathway [29].

KLF4 is a transcription factor that is one of the four Yamanaka pluripotency factors along with c-Myc, Sox2, and Oct4, which are capable of transforming somatic cells into induced pluripotent stem cells (iPS) [30,31]. KLF4 is a member of the Kruppel-like family of transcription factors that regulate proliferation, differentiation as well as stemness in embryonic stem cells [32–36]. While KLF4's ability to regulate pluripotency has been demonstrated, how this is mediated is not yet well defined. In stratified normal epithelia, KLF4 is also a regulator of late differentiation markers such as loricrin, filaggrin as well as components of the epithelial granular layer that contribute to the development of the cornified envelope [29,37,38]. Genetically engineered KLF4 knock out mice develop an immature skin with a fragile cornified envelope and dysfunctional fat layer incapable of providing barrier function leading to death within 12 hours after birth [37]. Given the diverse roles of KLF4 in maintaining basal cell characteristics while also regulating differentiation, we investigated which of its activities was important for the HPV lifecycle. Our studies demonstrate that KLF4 is critical for regulating late viral events upon differentiation. KLF4 levels are increased in HPV-positive cells through post-transcriptional mechanisms involving down regulation of miR-145 along with suppression of post-translational modifications that negatively affect its activity. These changes result in altered expression of KLF4 target genes in HPV-positive cells that are distinct from those seen in normal keratinocytes but necessary for regulating the differentiation-dependent HPV life cycle.

## Results

### KLF4 protein levels are elevated in HPV-keratinocytes

**Fig 1:**
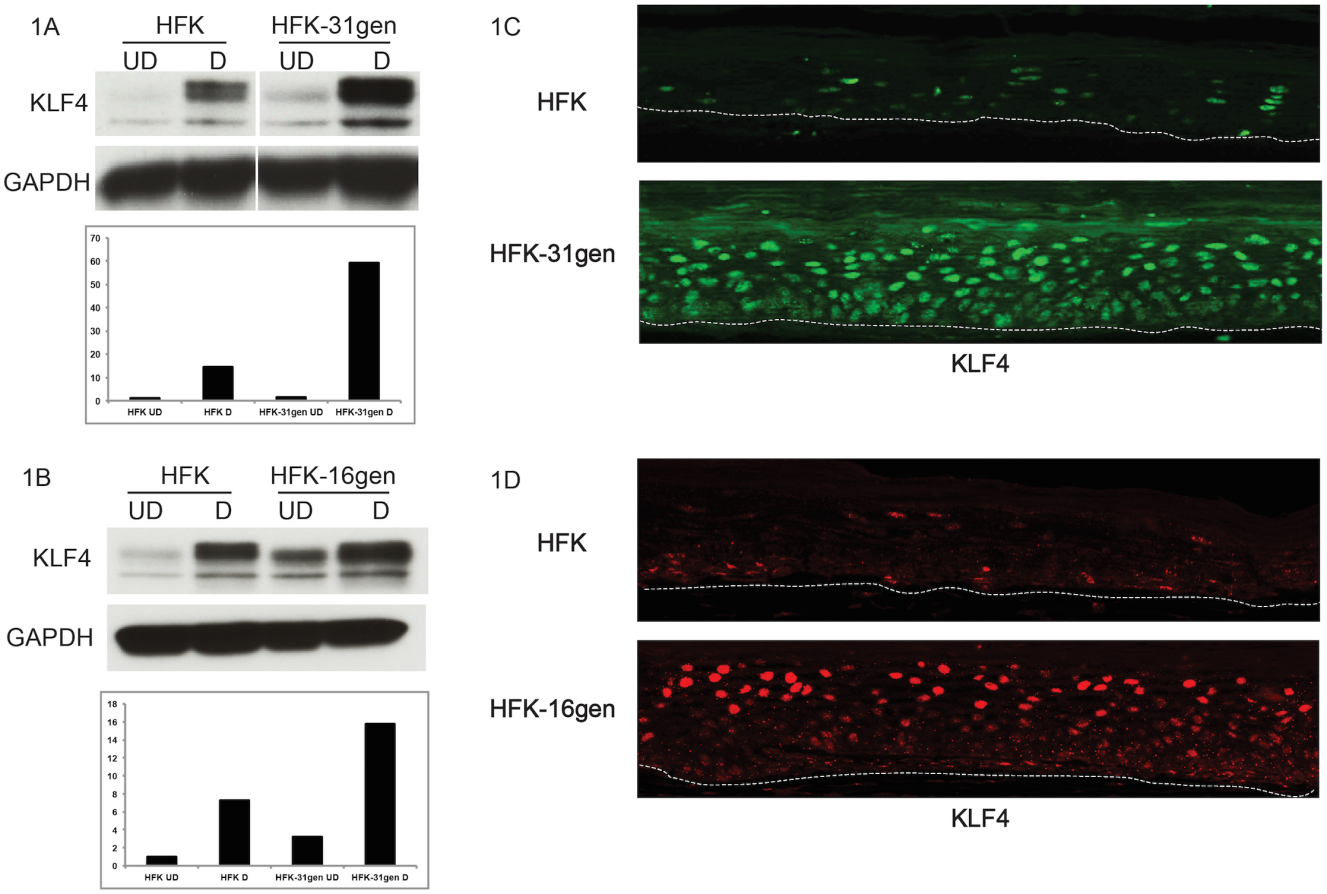
KLF4 protein levels are increased in HPV-31 and HPV-16 positive keratinocytes. Western blot showing the levels of KLF4 between genetically matched normal (HFK) and HPV-31 (HFK-31gen) / HPV-16 (HFK-16gen) keratinocytes. KLF4 protein levels were higher in both HPV-31gen (1A) and HPV-16gen (1B) cells in undifferentiated (UD) as well as differentiated conditions (D) induced by suspension in methylcellulose when compared to HFKs. GAPDH levels served as loading control. (1C and 1D) Immunofluorescence of KLF4 proteins in organotypic raft cultures. Matched HFK and HFK-31gen/HFK-16gen cells were grown as organotypic rafts for 13 days, formalin fixed and processed for KLF4 staining. Both HFK-31gen (1C) and HFK-16gen rafts (1D) stained strongly for KLF4 when compared to HFK rafts. Dotted lines mark the basal keratinocyte layer.

In undifferentiated normal foreskin keratinocytes (HFKs), the levels of KLF4 are low and increase substantially upon differentiation. A comparison of KLF4 levels in HFKs and HKF-31gen (HFKs from the same host background that had been stably transfected with re-circularized HPV-31 genomes) demonstrated higher levels in undifferentiated HFK-31gen cells and this further increased upon differentiation (Fig 1A), which is consistent with previous observations [26]. Similar increases in KLF4 levels were also seen in matched sets of normal and HFK-16gen (HPV-16 stably transfected HFKs) (Fig 1B). These same matched sets of cells were also grown as organotypic raft cultures and analyzed by immunohistochemistry for KLF4 levels and distribution. KLF4 was found to be expressed at higher levels in both HFK-31gen and HFK-16gen rafts compared to matched HFK controls (Fig 1C and 1D). In both HFK-31gen and HFK-16gen rafts, KLF4 levels were highest in suprabasal layers as compared to basal cells, which showed minimal staining. In HFK rafts, KLF4 staining was less distinct and more uniformly distributed.

### KLF4 is essential for progression of the HPV life cycle

**Fig 2:**
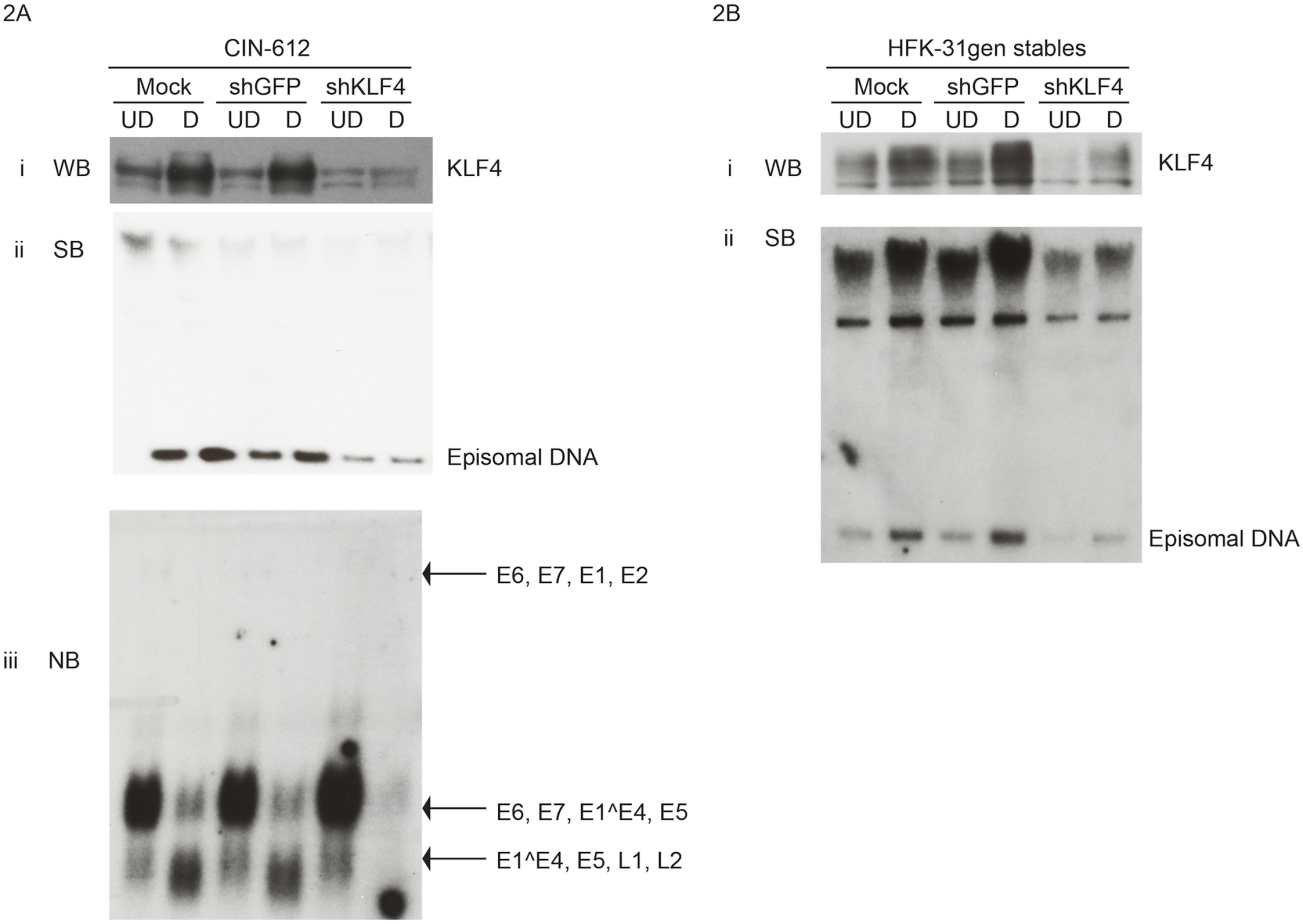
KLF4 is required for HPV DNA amplification and late gene expression. 2A. KLF4 was transiently silenced in CIN-612 cells using lentiviral shRNAs. Differentiation was induced by suspending cells in methylcellulose [7,40]. (i) The reductions in KLF4 protein levels were observed by western analysis in both undifferentiated and differentiated conditions of shKLF4 cells compared to mock and shGFP controls. (ii) Silencing KLF4 with shRNAs impaired the ability of the cells to amplify episomal DNA upon differentiation as shown by Southern blot analysis. (iii) Silencing KLF4 also resulted in the reduction in levels of late mRNA transcripts upon differentiation when compared to the controls as shown by northern blot analysis. 2B. KLF4 was stably silenced in HFK-31gen cells using lentiviral shRNAs. Following infection with shRNA lentiviruses, cells were expanded and selected with puromycin. (i) KLF4 protein levels were reduced in cells stably transduced with shKLF4 lentivirus compared to both mock and shGFP lentivirus controls as shown in the western blot. (ii) Stable silencing of KLF4 reduced the amount of viral DNA amplification upon differentiation compared to the controls as shown by Southern blot analysis.

To investigate if KLF4 had any role in regulating the HPV life cycle, lentiviruses expressing shRNAs against KLF4 were used to transiently infect CIN-612 cells, which are derived from a cervical biopsy and stably maintain HPV-31 episomes without expressing drug resistance markers[39]. For this analysis, we tested a series of KLF4 shRNAs and identified shRNAs that efficiently reduced KLF4 protein levels and pooled three of these shRNAs for further analysis. KLF4 levels were reduced following infection with pooled shKLF4 lentiviruses compared to infection with mock and shGFP controls (Fig 2A.i). Cells were then examined by Southern blot analysis for stable viral replication in monolayer cultures, and for viral DNA amplification following differentiation in methylcellulose. Cells in which KLF4 levels were reduced exhibited minimal change in episome levels in undifferentiated cells and episomes failed to amplify upon differentiation. In contrast, cells transduced with shGFP lentiviruses displayed viral DNA amplification upon differentiation similar to non-transduced (mock) controls (Fig 2A.ii). Knockdown of KLF4 also resulted in a severe impairment in late viral transcript levels as measured by northern blot analysis (Fig 2A.iii). To rule out the possibility that off target effects are responsible for inhibiting viral DNA amplification, we infected CIN-612 cells individually with the three different shRNAs that target different regions of the KLF4 gene. Following infection, we observed that each of these shRNAs individually blocked HPV amplification indicating that the effects are specific for KLF4 (S1 Fig). We also checked the mRNA levels of other KLF family members including KLF-3, 5, 14, and other members by RNA sequencing, which remained unaltered upon pooled KLF4 shRNAs mediated silencing. To investigate the long-term effects of KLF4 knockdown, HFK-31gen cells were transduced with shRNA lentiviruses and expanded in culture containing puromycin. Reductions in KLF4 levels in these stable cell lines were confirmed by western blot analysis (Fig 2B.i) Cells in which KLF4 was knocked down grew at rates similar to controls, exhibited a modest reduction in the levels of episomes in undifferentiated cells and failed to amplify upon differentiation (Fig 2B.ii). These results demonstrate that KLF4 provides critical functions for the HPV life cycle, primarily in differentiated cells.

### KLF4 binds to the HPV URR and regulates viral gene expression

**Fig 3:**
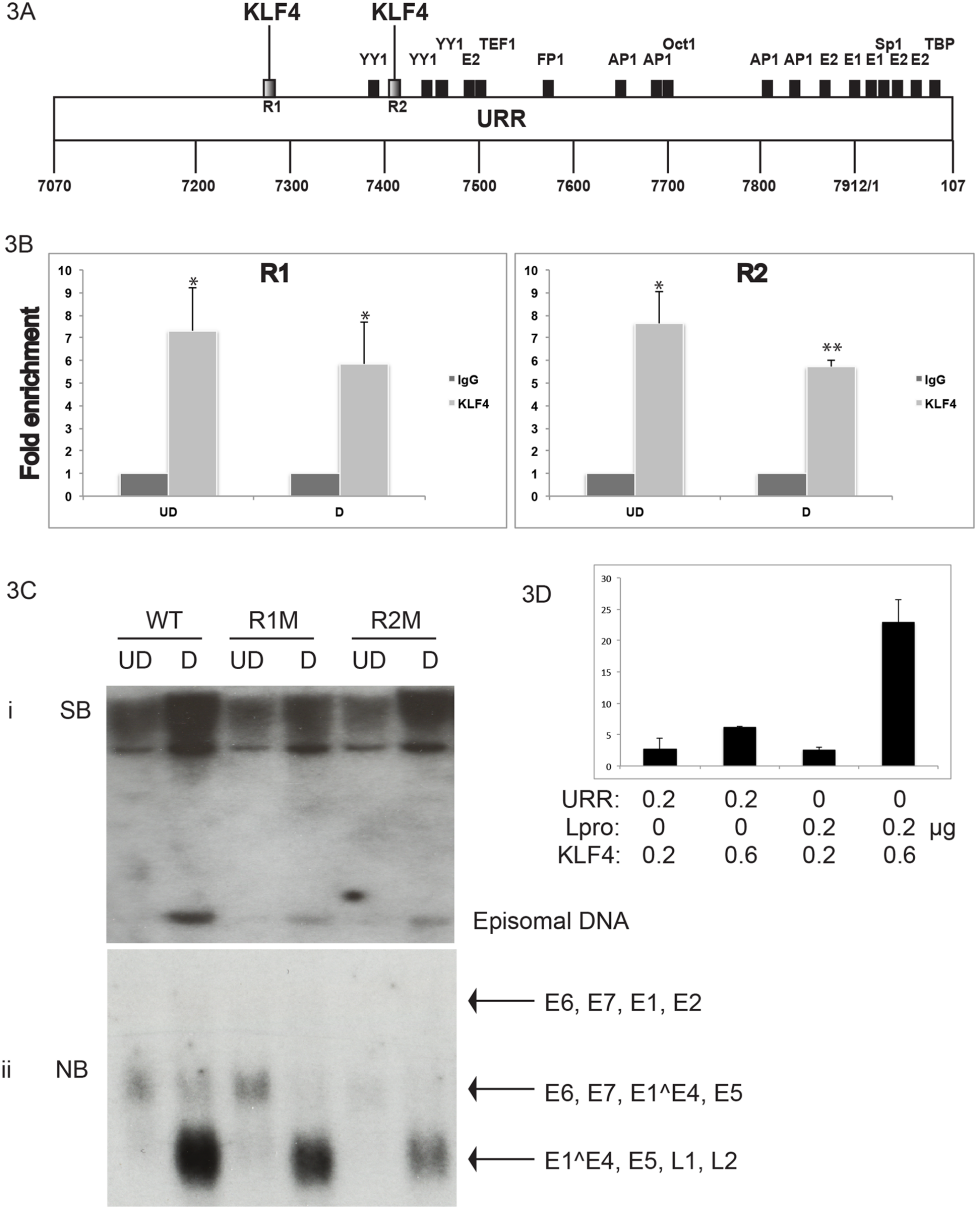
KLF4 binds to the Upstream Regulatory Region (URR) of HPV-31 and activates viral promoter reporter constructs. (3A). HPV-31 Upstream Regulatory Region is represented as a linear schematic with potential transcription factor binding sites marked in black rectangles. KLF4 consensus binding regions: region1 (R1) and region2 (R2) are marked as patterned black rectangles. (3B). The two KLF4 binding regions in the viral URR were analyzed for KLF4 binding in CIN-612 cells using Chromatin Immunoprecipitation (ChIP) assay. Binding of KLF4 to both URR regions was significantly enriched over IgG controls in both undifferentiated (UD) and differentiated (D) conditions (3B). p values: *<0.01, **<0.001. (3C). Single nucleotide changes were introduced into the whole HPV-31 genome, HFKs transfected with wildtype and mutant genomes and stable cell lines selected. The stably transfected cells were designated as R1M (region 1 of URR) and R2M (region 2 of URR). (i) Both R1M and R2M cells displayed impaired viral DNA amplification upon differentiation compared to the wild type cells as shown by Southern blot analysis. (ii) Both mutants produced significantly reduced late transcripts upon differentiation compared to the wild type cells as shown in the northern blot. (3D). KLF4 expression plasmid was co-transfected with either URR- or Lpro- (late promoter) luciferase reporter plasmids into 293T cells, and relative luciferase activities were measured. KLF4 activated luciferase activity of both URR and Lpro constructs at both low and high concentrations.

KLF4 is a transcription factor that binds to CACCC consensus sequences to regulate gene expression [29,34,38,41]. The HPV-31 Upstream Regulatory Region (URR) contains the viral origin of replication as well as binding sites for transcription factors that regulate viral gene expression. The HPV-31 URR also contains two KLF4 binding sites designated as R1 and R2 (regions 1 and 2). Using Chromatin Immunoprecipitation (ChIP) assays, we first determined that KLF4 binds to both R1 and R2 of HPV-31 URR to comparable levels in both undifferentiated and differentiated conditions and which are consistently higher than IgG controls (Fig 3B). We also showed that KLF4 did not bind to GAPDH and 18srDNA sequences (S2 Fig) proving that KLF4 binding to the URR regions is specific. To determine if these two KLF4 binding sites in the HPV-31 URR have any functional significance for the HPV life cycle, individual point mutations were introduced into the viral genome using site directed mutagenesis and verified by whole genome sequencing. Stable cell lines were generated by transfection with wildtype, region 1 URR mutant (R1M), and region 2 URR mutant (R2M) recircularized HPV 31 genomes, and expanded. Mutations in either region of the URR led to significantly reduced levels of viral DNA amplification as shown by Southern blot analysis (Fig 3Ci) along with impaired viral late gene expression as indicated by northern blot analysis (Fig 3Cii) upon differentiation. These results suggest that KLF4 acts a positive regulator of viral gene expression. To further confirm a role of KLF4 in activating viral expression, KLF4 expression plasmids were co-transfected with either URR-Luciferase or Lpro-luciferase (URR containing Late promoter) plasmids into 293T cells. KLF4 activated both URR-and Lpro-luciferase activities at low levels of transfected expression vector (0.2μg) and the activity further increased at higher levels (0.6 μg) of KLF4 expression vector (3D). While overexpression of KLF4 in transient assays can activate both early and late reporters, in viral infections KLF4 are low in undifferentiated cells and high in differentiated layers suggesting KLF4’s primary effect may be to regulate the late viral promoter upon differentiation.

### KLF4 is important for regulating cyclin and loricrin levels in suprabasal layers

**Fig 4:**
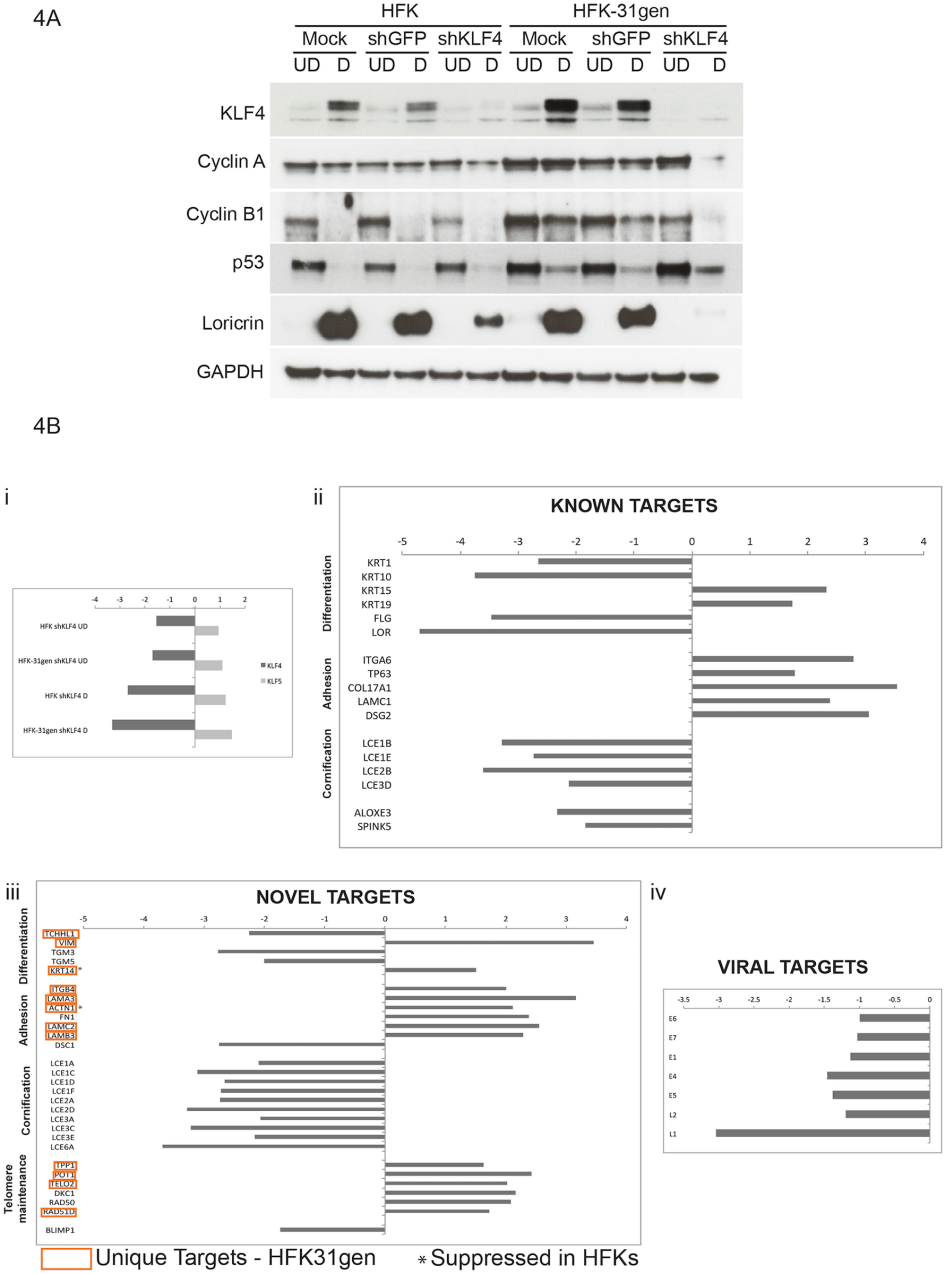
KLF4 regulates a distinct set of cellular targets between HPV-31 and normal keratinocytes. (4A). Genetically matched sets of HFK and HFK-31gen cells were transiently silenced for KLF4 using lentiviral transduction and were subsequently analyzed for known cellular targets of KLF4 using western blot analysis. KLF4 silencing was comparable between the two cell types as represented in the top panel. Silencing KLF4 in HFK-31gen cells had significant reductions in the levels of cyclin A, cyclin B1 and loricrin and an increase in p53 levels. In contrast, these changes were not observed in HFKs as evident from comparable levels of cyclin A, cyclin B1 and p53 between control and KLF4 silenced HFKs. Loricrin levels were modestly reduced by KLF4 silencing in HFKs as compared to HFK-31gen cells, where a complete loss of expression was detected. GAPDH served as a loading control. (4B). RNA isolated from the same cells as in (4A) was subjected to RNA-seq analysis. (i) KLF4 knock down was confirmed and was comparable between HFKs and HFK-31gen samples in both undifferentiated and differentiated conditions. Levels of related KLF5 did not vary upon KLF4 silencing. (ii, iii) Differentially expressed targets are represented as fold differences upon KLF4 silencing in differentiated HFK-31gen cells over HFKs. These differentially expressed targets were categorized into known and novel KLF4 targets. Targets under known and novel categories were further separated based on their known biological functions. (iii) Targets specifically regulated only in HPV-positive HFK-31gen cells, while unchanged in HFKs are marked with red boxes. Targets such as KRT14, and ACTN1 were suppressed in HFKs but activated in HPV-positive cells and are highlighted with asterisks. (iv) Viral targets of KLF4 are represented as fold reduction upon KLF4 silencing in HFK-31gen cells compared to shGFP controls.

KLF4 regulates differentiation as well as proliferative capability in basal/stem-like cells [33,37,42–45]. We investigated if these two pathways were targeted in HPV-positive cells by screening for changes in cell cycle regulatory genes such as the cyclins, along with differentiation-specific markers such as loricrin. Genetically matched HFKs and HFK-31gen cells were infected with shKLF4 lentiviruses and total protein lysates were examined for levels of KLF4, cyclins A and B1, and loricrin by western blot analysis. These assays showed that KLF4 levels were reduced to comparable levels in both HFK-31gen cells and HFKs following transduction with lentiviruses expressing KLF4 shRNAs (Fig 4A). By maintaining high levels of cyclins A and B1, HPV-positive cells remain active in the cell cycle upon differentiation to allow for viral DNA amplification. Upon differentiation of HPV-positive cells, the levels of cyclin A were higher in comparison to those seen in HFKs. Knockdown of KLF4 with shRNAs significantly reduced cyclin A levels in differentiated HFK-31gen cells while only a modest reduction as seen in HFKs. Consistent with previous observations [40], cyclin B1 levels were maintained at high levels in HFK-31gen cells upon differentiation but not in HFKs, and these levels were reduced when KLF4 was knocked down with shRNAs. Furthermore, the levels of the differentiation-specific protein loricrin were comparable in HFKs and HFK-31gen cells, but knockdown of KLF4 had a greater effect in reducing loricrin in HFK-31gen cells (Fig 4A). These experiments indicate that KLF4 regulates genes involved in cell cycle control and differentiation in HPV-positive cells, and that these functions of KLF4 are most significant in HPV-positive cells as compared to HFKs.

### Global transcriptional targets of KLF4 in HPV-positive keratinocytes

KLF4 is a transcription factor that regulates the expression of a number of cellular genes [29,34,38]. To investigate if this regulation was altered in HPV-positive cells, we performed global RNA-seq analyses on HFKs and HFK-31gen cells that had been transduced with control (shGFP) and shKLF4 lentiviruses. The use of KLF4 knockdowns for this assay was critical as it allowed us to identify which genes are transcriptional targets of KLF4. In this analysis, cells were infected with lentiviruses expressing shRNAs and after 48 hours were cultured in methylcellulose for an additional 48 hours to induce differentiation. RNA-seq analyses were then performed on mRNAs from both undifferentiated and differentiated cells. We first confirmed that KLF4 mRNA levels were reduced in cells transduced with KLF4 shRNA expressing lentiviruses, and found reductions to similar levels in both HFKs and HFK-31gen cells compared to shGFP controls (Fig 4B i). As a control we examined the levels of the related KLF5 mRNAs and saw no difference in mRNA levels between cell types after KLF4 silencing as compared to shGFP controls (Fig 4B i). To identify genes regulated by KLF4 we applied a threshold criteria of at least 1.5 fold change following KLF4 knockdown, along with a minimum of 100 reads to eliminate genes expressed at low levels. The complete set of KLF4 target genes in HFKs and HFK-31gen cells are presented in S1 Table. Genes that were differentially expressed between these cells types upon KLF4 silencing were categorized based on their functions and represented as fold activation or suppression in HFK-31gen cells over HFKs (Fig 4B). Our studies indicate that many previously identified KLF4 genes were either enhanced in activation or suppression by 2 to 5 fold in HPV-positive cells. Included among previously known KLF4 responsive genes whose expression was significantly altered in HPV-positive cells were genes associated with differentiation, factors involved in cell–cell/cell-matrix adhesion, and those associated with cornified layer formation (Fig 4B ii, S4 Fig). The changes in KLF4 target gene expression between HFK and HFK-31gen control cells (shGFP) are shown in S3 Fig. Differentiation associated genes such as trichohyalin, filaggrin, keratin 5, keratin 14 are all increased two to five fold in HPV-positive cells, while genes involved in cell adhesion, such as laminin alpha 3, laminin gamma 2, desmocolin 1, vimentin, and collagen17 alpha 1, are decreased to 2 to 5 fold greater compared to HFKs. In addition to previously characterized targets of KLF4, we identified a number of uncharacterized KLF4 targets including genes associated with differentiation, cell adhesion, telomere maintenance, and DNA damage (Fig 4B iii, S4 Fig). Importantly, we identified a series of novel genes (highlighted in red in Fig 4B iii) that are uniquely regulated by KLF4 in HPV-positive HFK-31gen cells but not in HFKs. This latter group includes trichohalin-like1, whose expression is enhanced by over three fold, as well as genes such as vimentin, Laminins (alpha3, beta3, gamma2), Actinin1, Protection of telomeres 1, and Telomere maintenance 2, which are suppressed by KLF4 up to 2 to 3 fold in HPV-positive cells. A subset of genes that were activated rather than suppressed by KLF4 in HFK-31gen cells as compared to HFKs is listed in S5 Fig. Finally, we identified late viral transcripts, particularly those encoding L1, to be KLF4 targets. KLF4 knockdown had a minimal effect on early transcripts, while late transcripts encoding L1, E4, and E5 were significantly reduced (Fig 4B iv).

### Blimp1 is a novel target of KLF4, which binds to KLF4 and activate viral transcription

**Fig 5:**
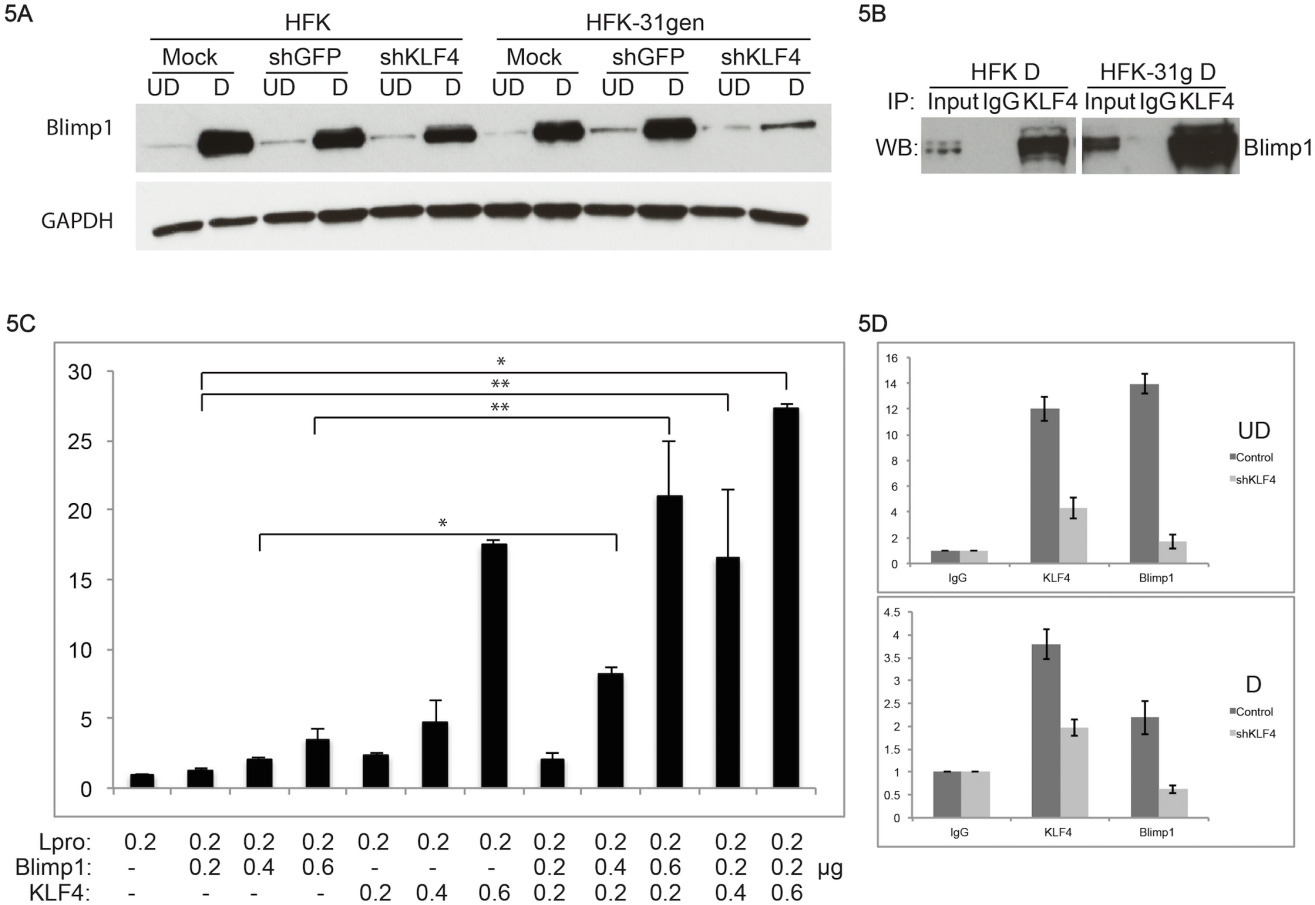
Blimp1 is a target of KLF4 that binds to KLF4 and activates viral transcription. (5A) Protein lysates from KLF4-depleted HFKs and HFK-31gen cells were analyzed for Blimp1 levels. Blimp1 protein levels were reduced in both KLF4-silenced HFKs and HFK-31gen cells with the most significant effect observed in the latter cells. (5B) Co-immunoprecipitation assays were performed in differentiated HFKs and HFK-31gen cells by immunoprecipitation with KLF4 antibodies and probing complexes for Blimp1 by western blot analysis. KLF4 bound to Blimp1 was significantly enriched in HFK-31gen lysates compared to HFK lysates. (5C) Blimp1 expression plasmid was co-transfected with Lpro-luciferase plasmid into 293T cells either in the presence or absence of KLF4 expression plasmids. Blimp1 alone modestly increased luciferase activity but upon co-transfection with KLF4 significantly increased luciferase activity in a dose-dependent manner. (5D) Chromatin immunoprecipitation assays were performed using control HFK-31gen and HFK-31gen-shKLF4 stable cells. Blimp1 bound to KLF4 binding site R2 in the control cells, whereas the Blimp1 binding ability decreased in shKLF4 cells.

One additional target of KLF4 identified by our analysis in HPV-positive cells is Blimp1. Blimp1 was recently identified as a factor that cooperates with KLF4 in EBV infected keratinocytes to regulate viral gene expression, as well as lytic replication in suprabasal layers [46]. Our RNA-seq analysis indicated that Blimp1 mRNA levels were reduced upon silencing of KLF4 in both HFKs and HFK-31gen cells, with approximately two fold greater reduction in HFK-31gen cells compared to HFKs (Fig 4B iii). To confirm that Blimp1 is a KLF4 target, we examined protein lysates from KLF4-silenced HFKs and HFK-31gen cells for Blimp1 expression. Silencing KLF4 significantly reduced Blimp1 protein levels in HFK-31gen cells compared to HFKs (Fig 5A) reflecting RNA-seq data. We next used co-immunoprecipitation to determine if KLF4 forms protein complexes with Blimp1. KLF4 was found to bind to Blimp1 in both differentiated HFKs and HFK-31gen cells, with significantly higher levels in the latter cells (Fig 5B). It was next important to determine if Blimp1 cooperates with KLF4 in the activation of HPV promoters. For this analysis, HPV late promoter-luciferase reporters were co-transfected together with KLF4 and Blimp1 expression vectors in various ratios and screened for levels of luciferase expression. These late promoter luciferase reporters contain nucleotides 7045 through 891 of HPV31 genome, which includes the major start site for the late promoter (p742) as well as the complete URR. While Blimp1 expression alone had a modest effect on the activation, when co-transfected with KLF4, increased activation of luciferase expression in a concentration-dependent manner was seen (Fig 5C). We conclude that Blimp1 is a KLF4 target that forms protein complexes with KLF4 to additively activate HPV promoters. To verify if KLF4-Blimp1 association is important for the activation of HPV late gene expression, we conducted ChIP assays using HPV31 keratinocytes in which KLF4 was stably depleted with lentiviral shRNAs to examine Blimp1 binding to the KLF4 binding site R2 in the URR. Both KLF4 and Blimp1 bound to R2 in both undifferentiated and differentiated conditions. Upon stable knockdown of KLF4 with shRNAs, Blimp1 binding to R2 was significantly reduced both in undifferentiated and differentiated conditions (Fig 5D). These results indicate that KLF4 is required for Blimp1’s ability to bind efficiently to the HPV31 promoter.

### KLF4 functions alter in HPV-positive keratinocytes

**Fig 6:**
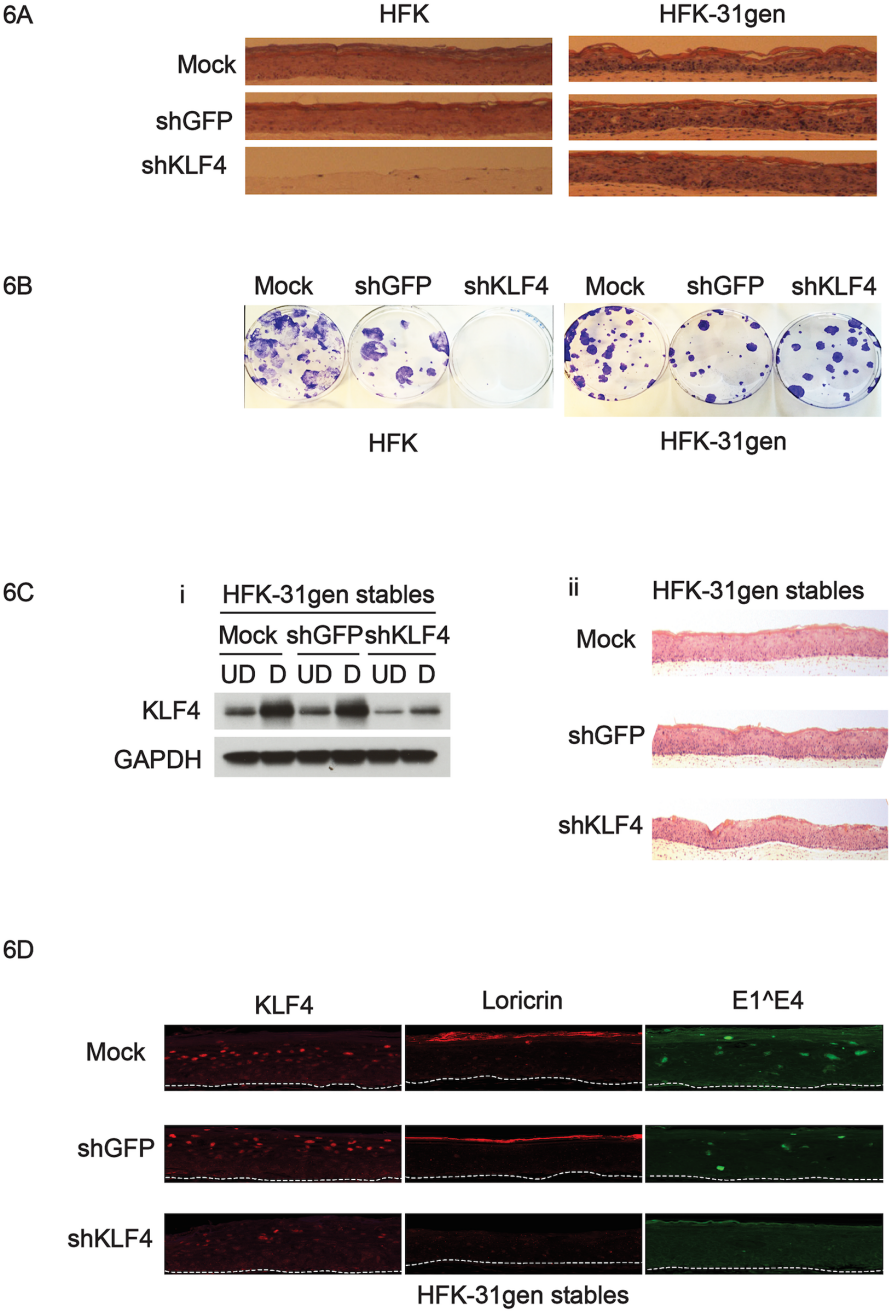
Altered functions of KLF4 in HPV-positive keratinocytes. (6A). Genetically matched sets of HFK and HFK-31gen cells were transiently silenced for KLF4 using lentiviral transduction and were subsequently analyzed for their ability to grow as organotypic rafts. KLF4 silenced HFK-31gen cells grew as rafts with morphologically altered cornified envelope, whereas KLF4 silenced HFKs did not form rafts. (6B). KLF4 silenced HFKs did not form any colonies in holoclone assay compared to controls, whereas KLF4 silenced HFK-31gen cells formed colonies comparable to controls. (6C. i) KLF4 is stably silenced in HFK-31gen cells. KLF4 protein levels were reduced in both undifferentiated and differentiated conditions compared to controls as shown in the western blot. (ii) Stable KLF4 silenced HFK-31gen cells formed rafts with morphologically altered cornified envelope. (6D) Raft sections were analyzed by immunofluorescence for KLF4, loricrin, and E1^E4. KLF4 silenced HFK-31gen rafts showed reduced KLF4 and no loricrin or E1^E4 staining compared to controls.

The data described above indicate that KLF4 has enhanced transcriptional activation and suppression abilities in HPV-positive cells compared to normal keratinocytes, suggesting it may provide different functions in these cells. We next investigated the effects of silencing KLF4 on cell growth and differentiation capabilities in HFKs and HFK-31gen cells. Cells were infected with shRNA lentiviruses, and 48 hours after transduction seeded onto collagen plugs and grown as organotypic raft cultures. Reductions in KLF4 protein levels were comparable in both sets of cells. Transient silencing of KLF4 in HFKs abolished the ability of cells to form stratified cultures in organotypic rafts, whereas KLF4-depleted HFK-31gen cells formed rafts with stratified layers but with morphologically altered cornified envelopes (Fig 6A). Similar results were seen in three independent experiments. We surmised that the inability of KLF4-depleted HFKs to form rafts might be due to a loss in stem cell proliferative capacity. To test this hypothesis, HFKs and HFK-31gen cells were assessed for their colony forming abilities using a holoclone assay [47]. In this assay, keratinocytes are seeded sparsely (100–500 cells) in 100 mm dishes, thereby forcing them to undergo multiple cell divisions. In such stringent conditions, only cells with extensive proliferation capacity such as stem cells and early stage transit-amplifying cells can form viable colonies, while late stage transit-amplifying cells produce abortive colonies due to their limited proliferative capacity. KLF4-depleted HFK-31gen cells formed colonies comparable to control cells, while KLF4-depleted HFKs completely abolished colony-forming abilities indicating a loss in stem cell-like proliferative activity (Fig 6B).

In a similar manner, it was not possible to generate stable cell lines of HFKs following infection with lentiviruses expressing KLF4 shRNAs, whereas both HFK-31gen and HFK-16gen cells readily formed lines with stable KLF4 depletion (Fig 6C, S6 Fig). HFK-KLF4 knockdown cells continued to proliferate for up to one passage following transduction but failed to expand after passaging. In contrast, HFK-31gen KLF4 knockdowns could be readily passaged and grew at rates comparable to parental cells. Reductions in KLF4 levels were confirmed in the HPV-positive cells after multiple passages by western blot analysis (Fig 6C.i). As observed in transient silencing experiments, KLF4-silenced HFK-31gen cells formed stratified cultures in organotypic rafts, but with morphologically altered cornified layers (Fig 6C.ii). Raft sections were further analyzed for KLF4, loricrin, and E1^E4 levels by immunohistochemistry. Loricrin and E1^E4 were used as a read out for the changes in cornified envelope composition and viral late gene expression respectively. KLF4 levels were substantially reduced in shKLF4 rafts compared to control rafts, and both loricrin and E1^E4 were not detected in KLF4-silenced rafts (Fig 6D). Similar effects were seen when KLF4 levels were silenced in HFK-16gen cells (S6A Fig), with morphologically altered cornified layers in organotypic raft cultures (S6B Fig). These results indicate that KLF4 may have similar functions in modulating cell cycle and differentiation capabilities of multiple high-risk HPV types. Furthermore, our studies indicate that KLF4 regulates the expression of genes in undifferentiated HFKs that are critical for proliferation, while similar effects are not seen in HPV-positive cells.

### Post-translational modifications of KLF4 alter in HPV-positive keratinocytes

**Fig 7:**
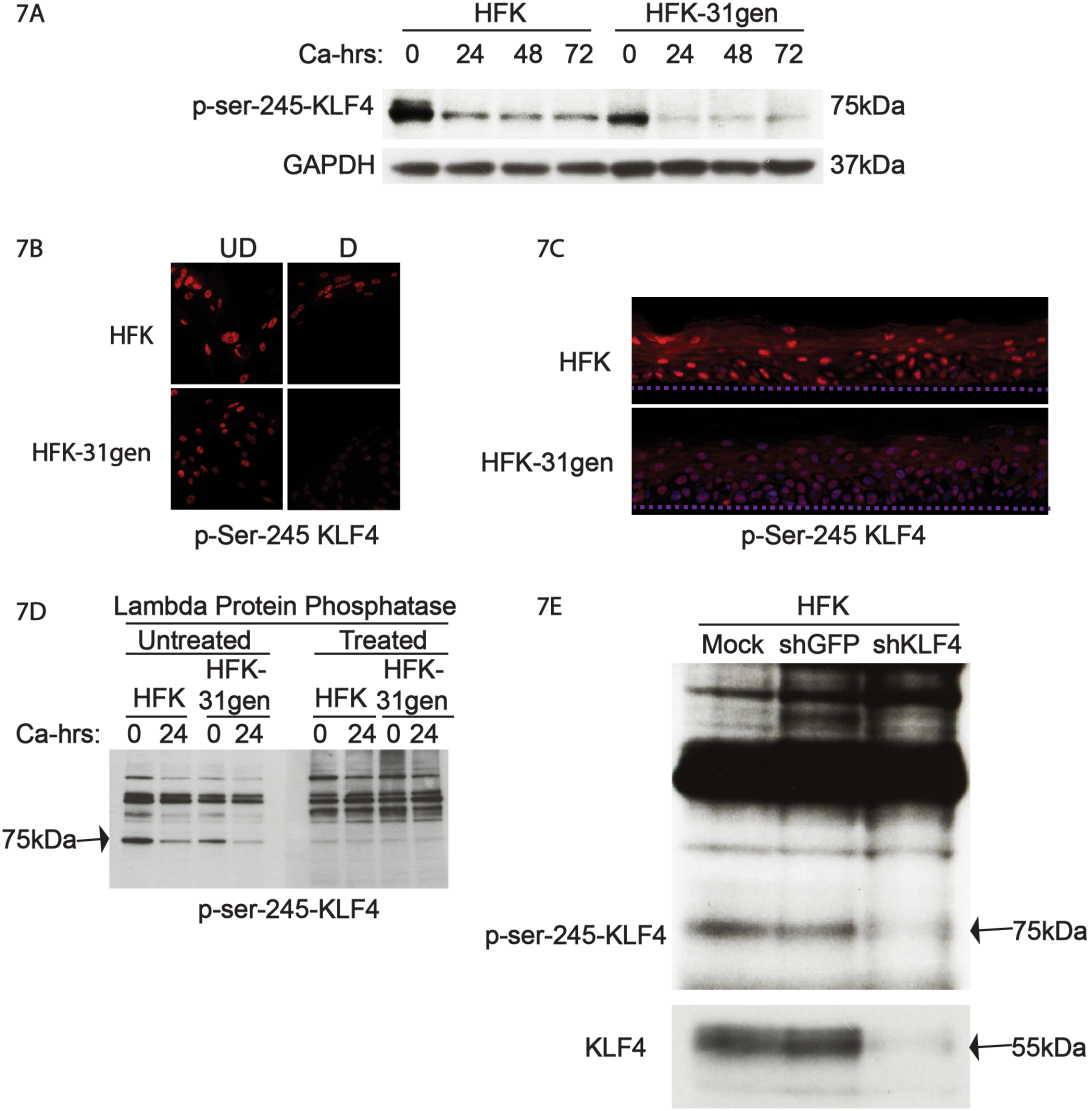
KLF4 is hypo-phosphorylated in HFK-31gen cells compared to HFKs. (7A). Genetically matched HFKs and HFK-31gen cells were grown in high calcium media to induce differentiation and protein levels of phospho-ser-245 KLF4 were analyzed by western blot analysis. Levels of p-ser-245 KLF4 were lower in HKF-31gen cells than HFKs in both undifferentiated (0 calcium) condition and differentiated high calcium conditions throughout the time course. (7B). Immunofluorescence analysis for p-ser-245 KLF4 also showed a similar trend to western results, where HFK-31gen cells showed less intense staining than HFKs in both undifferentiated and differentiated conditions. (7C). HFK-31gen rafts stained less intensely for p-ser-245 than HFK rafts. (7D). Protein lysates from the analysis shown in panel A were electrophoresed in SDS polyacrylamide gels, and western blot analysis performed in duplicate. These were then treated with either mock- or protein phosphatase and then analyzed with antibodies for p-ser-245 KLF4 levels. Phosphatase treatment specifically reduced the levels of the 75kDa band confirming that it is a phospho-specific band. (7E). Protein lysates from KLF4 silenced HFKs were analyzed for KLF4 and p-ser-245 KLF4 levels. Silencing KLF4 reduced KLF4 levels (bottom panel) as well as the 75kDa p-ser-245 band confirming that it is a KLF4-specific band.

The finding that KLF4 has different functions in HFKs and HPV-positive cells led us to investigate a potential mechanism, beyond changes in KLF4 expression levels, that could contribute to these effects. KLF4 undergoes post-translational modifications such as phosphorylation, sumoylation, and acetylation, which in turn determine its binding partners and ability to activate or suppress gene expression [43,48–51]. KLF4 is phosphorylated at serine 245 and this results in suppression of its transcriptional activities. We therefore investigated if there were any differences in phosphorylation between HFKs and HPV-positive cells as determined by western blot analysis. In undifferentiated HFK-31gen cells, the levels of phospho-Ser-245 KLF4 levels were substantially reduced as compared to matched HFKs. Furthermore, upon differentiation, p-KLF4 levels were also lower in HFK-31gen cells than HFKs (Fig 7A). Next, we used immunofluorescence analysis of HFKs and HFK-31gen cells grown on coverslips in either normal or high calcium media for 72 hours to screen for effects on the levels of p-KLF4. Less intense staining for p-KLF4 was observed in HFK-31gen cells as compared to HFKs in either undifferentiated or differentiated conditions (Fig 7B). Calcium-induced differentiation was used for these analyses as this method does not distort cell morphologies, as is seen with methylcellulose-induced differentiation. Finally, similar distributions and levels of p-KLF4 were detected in organotypic raft cultures of these same cells (Fig 7C).

Interestingly, the band we identified by western analysis as phospho-Ser-245 KLF4 migrated at approximately 75 kDa, which is approximately 20kDa higher than the unmodified KLF4 band. While phosphorylation alone does not induce such large shifts in mobility, phosphorylation coupled with sumoylation could induce such a shift. Phosphorylation is often a prerequisite for sumoylation and KLF4 has been reported to be sumoylated under some conditions. To provide support that this band was actually a phosphorylated form of KLF4, lysates from HFKs and HFK-31gen cells grown in both undifferentiated and high calcium conditions were run in duplicate on a single polyacrylamide gel. After electrophoresis, the gel was transferred to a PVDF membrane and the membrane was cut into two halves, which were either incubated with lambda phosphatase in buffer or buffer alone, for one hour. The membranes were then blocked and processed as usual for western analysis. Treatment with lambda phosphatase specifically reduced the levels of the 75kDa band, without altering the surrounding non-specific bands, demonstrating that this band is a phosphorylated protein (Fig 7D). To further demonstrate that the 75kDa band is specific to KLF4, protein lysates of HFKs transiently infected with lentiviruses targeting KLF4 were screened by western blot with antibodies against unmodified KLF4 and phospho-ser-245 KLF4. KLF4-specific shRNAs reduced levels of both unmodified KLF4 and the observed 75kDa phospho-band (Fig 7E) in comparison to control samples. These experiments verified that KLF4 is phosphorylated at Serine 245 and runs at approximately 75kDa.

**Fig 8:**
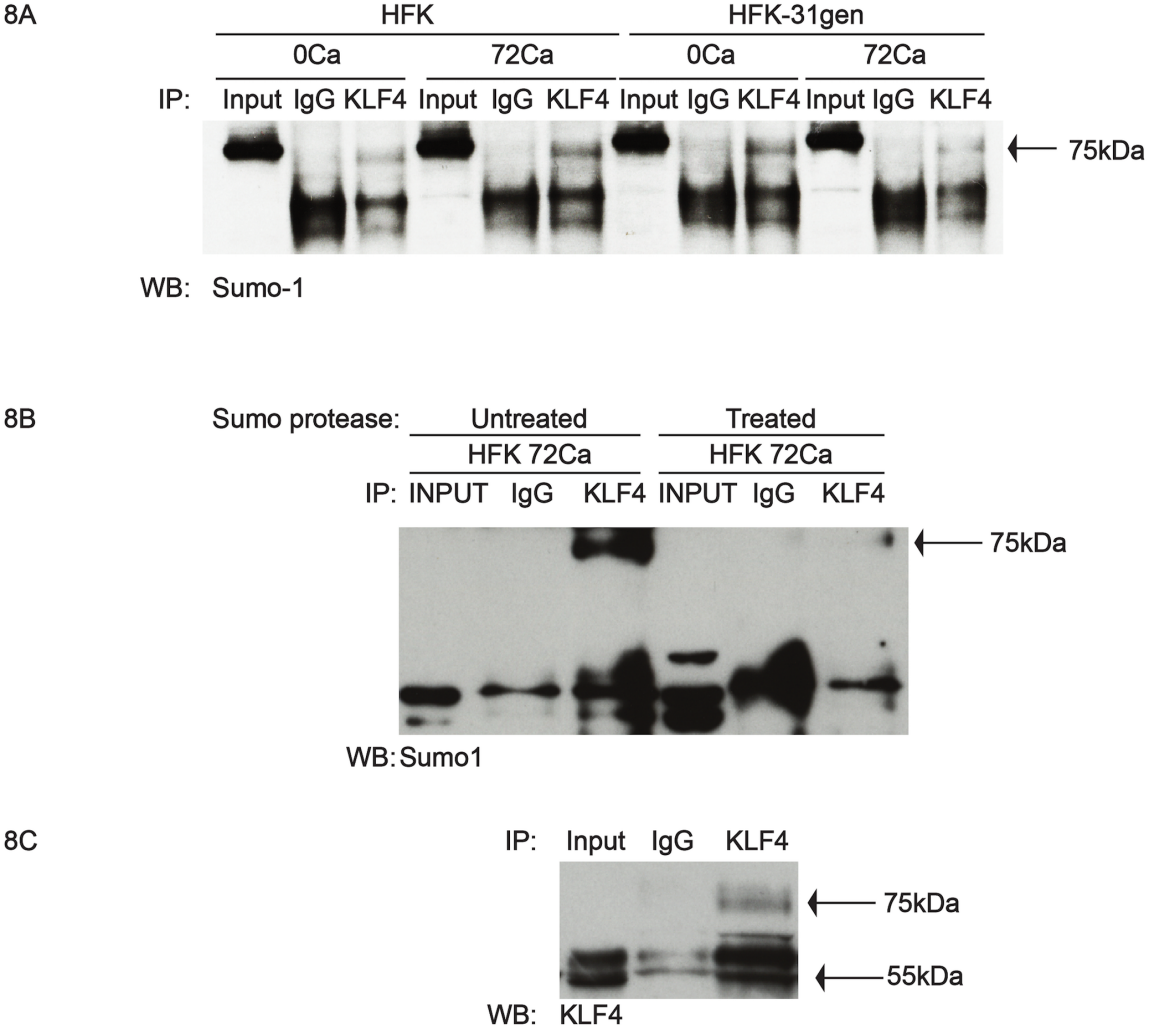
KLF4 is hypo-sumoylated in HFK-31gen cells compared to HFKs. (8A). Protein lysates from genetically matched HFKs and HFK-31gen cells grown in undifferentiated and differentiated conditions were immunoprecipitated with KLF4 antibodies and analyzed by western analysis using antibodies for Sumo1. In undifferentiated cells KLF4 was sumoylated (75kDa band) to comparable levels in both cell types, whereas sumoylated KLF4 levels were reduced in differentiated HFK-31gen cells compared to HFKs. The band in the input lane is slightly higher than the sumo-KLF4 band and corresponds to a Sumo1/RanGAP1 complex as identified by the antibody manufacturer (8B). Protein lysates from differentiated HFKs from the above experiment were treated with sumo protease or mock treated and immunoprecipitated as above. Treatment with sumo protease specifically reduced the 75kDa band confirming that KLF4 is sumoylated. (8C) Immunoprecipitation experiments using KLF4 pull down and KLF4 western to confirm the 75kDa band is KLF4. The 55kDa unmodified KLF4 protein is also seen.

We next investigated if the 75kDa band is a sumoylated form of KLF4. Protein lysates from matched HFKs and HFK-31gen cells grown in either low or high calcium media were immunoprecipitated with total KLF4 antibody and then screened for the presence of Sumo-1 by western analysis. KLF4 pull down samples yielded a 75kDa band, which was absent in IgG pull down samples. In addition, the intensity of the band was lower in HFK-31gen differentiated samples than HFKs, consistent with changes observed in the phosphorylation experiments (Fig 8A). To validate that the 75kDa band is sumoylated, we performed immunoprecipitation with either mock or sumo protease- treated protein lysates of differentiated HFKs. Immunoprecipitation of mock-treated samples with KLF4 antibodies resulted in the appearance of the 75kDa band, but the levels of this band were significantly reduced in sumo protease-treated KLF4 immunoprecipitated samples (Fig 8B). These experiments demonstrate that the 75kDa KLF4 band is both sumoylated and phosphorylated, which together explain the observed 20kDa shift in weight. We next wanted to confirm that the sumoylated band is indeed KLF4 and not any other KLF4 binding protein undergoing sumoylation. For this analysis, we performed immunoprecipitation assays using a mouse secondary KLF4 antibody to pull down protein complexes and a rabbit secondary KLF4 antibody for the western blot analysis. KLF4-KLF4 immunoprecipitation experiments demonstrated an enrichment of unmodified 55 kDa KLF4 along with its isoforms compared to igG control and more importantly showed an enrichment of a 75kDa KLF4 band (Fig 8C). These results indicate that the levels of phosphorylated and sumoylated KLF4 are significantly reduced in HPV-positive cells.

### HPV proteins E6 and E7 alter KLF4 expression and activity

**Fig 9:**
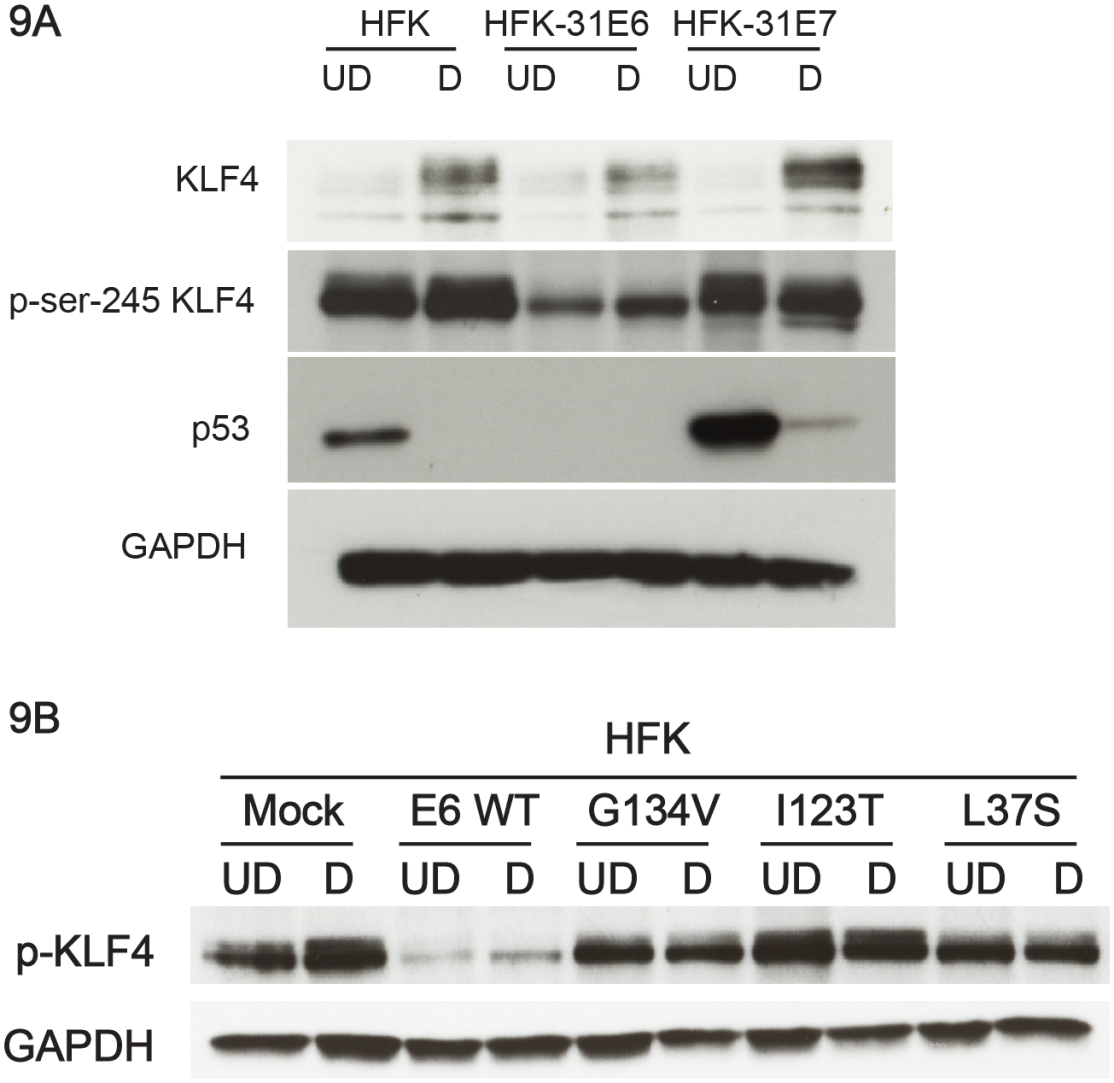
HPV proteins E6 and E7 alter KLF4 expression and activity. (9A). Protein lysates from genetically matched HFK and HPV31 E6 and E7 over expressing cells grown in undifferentiated and differentiated conditions were examined by western analysis for unmodified KLF4 and phospho-Ser-245. E7 lysates showed substantially increased levels of KLF4 compared to HFK controls while E6 lysates showed a modest decrease. KLF4 phosphorylation levels were significantly reduced in E6 lysates compared to HFK controls while E7 lysates did not show much alteration as compared to HFKs. Analysis for p53 levels served as a positive control for E6 and E7 transfections. GAPDH served as a loading control. (9B). Protein lysates from genetically matched HFK and HPV31 wild type E6 and E6 mutants over expressing cells grown in undifferentiated and differentiated conditions were examined by western analysis for phospho-Ser-245. Wildtype E6 reduced the levels of phospho-ser-245 KLF4 but none of the E6 mutants displayed any alterations in phospho-ser-245 KLF4 levels. GAPDH served as a loading control.

We next investigated which viral proteins are responsible for the increased levels of KLF4 and suppression of its post-translational modifications. For this analysis, we used retroviruses expressing either HPV-31 E6 or E7 to infect HFKs and to isolate stable cell lines. Lysates from both undifferentiated and differentiated cells were then screened by western analysis for total levels of KLF4 as well as phospho-Ser 245 KLF4. Total KLF4 levels were elevated in both E6-and E7-expressing keratinocytes under undifferentiated or differentiated conditions, with the largest effect mediated by E7 (Fig 9A). In contrast, the levels of phospho-Ser245 KLF4 levels were significantly reduced in E6-expressing cells in either undifferentiated or differentiated conditions (Fig 9). p53 levels were used as a surrogate marker for E6 and E7 expression, as p53 levels were significantly reduced in E6-expressing cells but increased in E7-cells [52]. We then wanted to check which one of E6’s functions is essential for the phosphorylation of KLF4. For this analysis, we generated stable cell lines of HFKs expressing previously characterized E6 mutants including E6 G134V (lack CBP/p300 binding) [53], E6 I128T (cannot degrade p53), and E6 L37S (cannot bind as well as degrade p53) [54] using retroviral vectors and checked for phospho-ser-245 KLF4 levels by western blot analysis. While wildtype E6 reduced the levels of phospho-ser-245 KLF4, none of the E6 mutants exhibited any alterations in phospho-ser-245 KLF4 levels (Fig 9B) indicating that E6’s ability to bind to p53 and p300 as well as degradation of p53 is essential for the reduction of phosphorylation of KLF4. To understand the mechanisms responsible for E7 mediated increases in KLF4 levels, we investigated a possible role for NFκB. Previously, we showed that E7 decreases miR-145 levels [26] and additional studies demonstrated that E7 reduces NFκB activity [55]. We observed a corresponding decrease in NFκB activity in HPV-positive cells (S7A Fig) and determined that the promoter of miR-145 contains two p65 (NFκB active subunit) binding sites. Using transient reporter assays, we further demonstrated that increasing levels of p65 expression vector activated miR-145 promoter activity in a dose-dependent manner (S7B Fig). This indicates that E7 may contribute to increased levels of KLF4 through NFκB inactivation, while E6 is primarily responsible for suppression of KLF4 phosphorylation.

## Discussion

High-risk human papillomaviruses infect keratinocytes and modulate both the expression and activity of host factors to control their differentiation-dependent productive life cycles [7,8,26,56,57]. Our studies identify KLF4 as a critical host transcription factor that regulates the HPV life cycle by controlling viral gene expression, cellular differentiation, and cell cycle capabilities in suprabasal epithelial layers. KLF4 is one of four Yamanaka pluripotency factors, which along with Oct4, SOX2 and c-Myc are able to reprogram somatic cells into pluripotent stem cells by targeting their proliferation and differentiation capabilities. In HPV-positive cells, E7 and E6 proteins regulate KLF4 levels and activity, respectively, through post-transcriptional and post-translational mechanisms to induce a number of activities distinct from those seen in normal epithelia. E7 controls KLF4 levels post-transcriptionally by suppressing the expression of a cellular microRNA, miR-145, which targets KLF4 transcripts, leading to increased levels of KLF4 proteins. The E6 protein acts through post-translational mechanisms to regulate KLF4 phosphorylation and sumoylation, which negatively affect KLF4 functions. These virally induced changes result in differential activation or suppression of previously characterized transcriptional targets of KLF4 as well as expression of novel genes, all of which contribute to cell proliferation, stratification and differentiation during the HPV life cycle.

The HPV life cycle is closely associated with differentiation of the infected host keratinocyte. Following infection of undifferentiated stem-like basal cells, viral genomes are established as low copy episomes that replicate coordinately with cellular chromosomes in S phase [2]. Upon differentiation, late viral events such as viral DNA amplification, late gene expression, and virion packaging are induced in differentiated suprabasal cells following transition into S/G2[58]. Since KLF4 is important for regulating stem-cell like proliferative abilities along with controlling differentiation, we investigated what role, if any, KLF4 played in either of these processes during the HPV life cycle.

In HPV-positive stratified epithelia, KLF4 is primarily expressed in suprabasal layers with only low levels in basal cells. Our studies demonstrate that KLF4 regulates late viral events in two ways in suprabasal cells. One function of KLF4 is to directly regulate expression of HPV late genes. Our studies show that KLF4 binds to two sites in the HPV-31 URR and that mutation of these sites reduces differentiation-dependent late gene expression as well as significantly impairs genome amplification. Similarly, silencing KLF4 with shRNAs impairs both viral late gene expression and viral DNA amplification. RNA seq analyses confirmed reductions in viral transcripts encoding L1, L2, E4, and E5 in HPV-positive cells in which KLF4 was knocked down with shRNAs, with minimal effects on early viral gene transcripts. In stable knockdown cultures, we observed a modest reduction in the levels of viral episomes, a significant impairment in genome amplification upon differentiation, and reduced late gene expression. Together, these data demonstrate a critical role for KLF4 in regulating late viral functions. Interestingly, one novel target of KLF4 is Blimp1 and it forms protein complexes with KLF4 to cooperatively regulate late viral transcription. The ability of KLF4 to act coordinately with Blimp1 to activate viral promoters has recently been shown to control EBV lytic replication[46]. Our studies along with those of Nawandar *et al*. demonstrate that KLF4 is a critical regulator of the differentiation-dependent life cycles of two oncogenic viruses, EBV and HPV. Whether KLF4 is post-transcriptionally regulated by EBV proteins, and if KLF4 controls Blimp1 expression in EBV-infected cells remains unclear.

In addition to a direct effect in regulating viral gene expression, RNA-seq analyses demonstrated that KLF4 positively regulates the expression of genes that control differentiation in the suprabasal cornified, granular, and spinous cell layers of HPV-positive keratinocytes. At the same time, KLF4 negatively regulates the expression of basal cell markers including cell-matrix adhesion genes, keratinocyte growth factor receptors, and telomere maintenance proteins. Not only were KLF4 functions enhanced in HPV-keratinocytes, but also a number of KLF4 targets unique to HPV-positive cells were identified including vimentin, integrin beta4, laminins, protection of telomeres1, and Rad51D.

Our studies show that in normal keratinocytes, KLF4 is required for the proliferative ability of basal cells and for the expression of differentiation genes following growth in raft cultures. Transient knockdown of KLF4 in normal keratinocytes reduced expression of both proliferation as well as differentiation associated genes, leading to a failure to grow beyond a single passage. Furthermore, these cells did not form colonies in holoclone assays nor grow in raft cultures. RNA-seq analysis demonstrated that KLF4 positively regulates expression of the cell-matrix adhesion gene Actinin1, which is critical for proliferation of basal cells as well as differentiation. In addition, we observed that KLF4 positively regulates expression of cytokeratin 14, which heterodimerizes with cytokeratin 5 to form the cytoskeleton of basal epithelial cells[59]. These changes may be responsible for the impaired proliferative ability of HFK stable KLF4 knockdowns, or additional effects may be required. Regardless, these functions are not critical in HPV-positive cells, which continued to propagate even after stable KLF4 knockdown. This difference could be the result of altered KLF4 and target gene expression in HPV- positive cells, or due to the actions of E6 and E7 in stimulating proliferation.

In HPV-positive cells, knockdown of KLF4 had minimal effects on the proliferative ability of undifferentiated cells, and stable knockdowns were readily established. The levels of Actinin1 and cytokeratin 14 are suppressed in HPV-positive cells unlike normal keratinocytes, which may contribute to their ability to survive and proliferate in long-term stable assays. In HPV-positive cells, KLF4 knockdown had its most significant effect upon differentiation: cells in which KLF4 was knocked down retained the ability to stratify in raft cultures but were significantly altered in the expression of differentiation specific genes and cell cycle regulators. The levels of loricrin and filaggrin, two differentiation genes associated with late viral functions, were significantly reduced. KLF4 silencing also severely affected the expression of many late cornified envelope (*LCE*) genes, leading to the formation of morphologically altered cornified layers in raft cultures. In addition, HPV-positive suprabasal cells were no longer active in the cell cycle, as evidenced by an absence of cyclin A or B1 proteins. While the levels of cyclin A and B1 proteins are reduced in KLF4 knockdowns, transcript levels are unchanged, indicating they are not direct transcription targets of KLF4. This indicates that KLF4 acts to regulate cyclin levels at the post-transcriptional level. While KLF4 silenced HPV keratinocytes formed stratified cultures in rafts, late viral functions were significantly impaired as evidenced by the loss of E1^E4 proteins. These analyses demonstrate that KLF4 has different critical functions in normal and HPV-positive keratinocytes.

Our studies show that HPV proteins regulate KLF4 levels and activities through post-transcriptional and post-translational changes. KLF4 levels are increased in HPV- positive cells, in part due to decreased levels of a cellular miRNA, miR-145, which we have previously shown to have recognition sites in the 3’UTR of KLF4 mRNA[26]. In addition, our previous studies suggest that E7 acts through NFκB to suppress miR-145 levels to increase KLF4 protein levels. Additional changes in KLF4 functions are likely the result of suppression of post-translational modifications such as sumoylation and phosphorylation. Sumoylation of KLF4 negatively regulates its activity and is dependent upon phosphorylation. KLF4 phosphorylation also negatively regulates KLF4 function facilitating its ubiquitination and subsequent enhanced degradation [43]. As discussed above, E7 suppresses miR-145 expression while E6 suppresses KLF4 phosphorylation and sumoylation. Erk1 and Erk2 kinases, which phosphorylate KLF4 to suppress its ability to induce proliferation and self-renewal in embryonic stem cells, may be potential targets of E6. Consistent with our observations, E6 has been shown increase the turnover of UBC9 leading to global reductions in sumoylation[60], providing a potential mechanism explaining its effect on KLF4. In addition to regulating protein stability, post-translational modifications can also regulate interactions with protein binding partners, cellular localization, and transactivation ability of factors. Suppression of KLF4 sumoylation or phosphorylation could induce the binding of different factors to KLF4, leading to activation of novel targets seen in HPV-positive cells.

In HPV-positive cells, KLF4 has a number of distinct functions that cannot be explained by increased levels alone. In particular, knockdown of KLF4 in normal keratinocytes blocks stratification while HPV-positive cells readily grow in raft cultures that have morphologically altered suprabasal layers. Furthermore, KLF4 knockdown in HPV-positive cells does not affect growth of basal cells in holoclone assays while this is inhibited in KLF4 knockdowns of HFKs. Finally, while many transcriptional targets of KLF4 are shared between HFKs and HPV-positive cells, there are a number of genes that are only repressed by KLF4 in HPV-positive cells while other genes that are activated by KLF4 in HFKs are repressed in HPV-positive cells. These differences cannot be easily explained by increases in KLF4 levels alone and implicate post-translational mechanisms as also being important.

Human cancer viruses regulate their productive life cycles by modulating the activities of cellular factors. Our work shows that high-risk human papillomaviruses alter the activities and expression of a critical cellular transcription factor, KLF4, through post-transcriptional and post-translational mechanisms, and thereby regulate differentiation-dependent late viral events. KLF4 has been shown to be a critical regulator of lytic replication of another oncogenic human cancer virus, EBV, but whether EBV alters KLF4 in a similar fashion is unclear. Furthermore, we show that the KLF4 transcriptome is altered in HPV- positive cells but whether similar changes occur in EBV cells remains to be determined. In summary, our work provides novel insight into mechanisms by which HPVs regulate host transcription factors to promote viral amplification and infection, and identifies a common pathway shared among human cancer viruses.

## Materials and Methods

### Ethics statement

The human keratinocytes used in this study were obtained from discarded foreskin circumcisions from anonymous donors by the Keratinocyte Core in the Northwestern University Skin Disease Research Center (SDRC) and are not classified as human subjects research. These specimens were not specifically collected for this study and lack all identifiers.

### Cell lines

Human Foreskin Keratinocytes (HFKs) were isolated from the foreskin tissues of neonates obtained from anonymous donors by the Keratinocyte Core in the Northwestern University Skin Disease Research Center (SDRC) and grown as described previously[8]. Recircularized HPV-31 genomes were transfected into HFKs along with antibiotic resistance plasmid pSV2 Neo and stably selected with G418 to obtain HFK-31gen cells. HPV-16 Cre genome containing plasmids were transfected along with Cre and pSV2 Neo plasmids into HFKs and stably selected with G418 to obtain HFK-16gen cells. CIN-612 cells are HPV31-positive cervical cells, which were obtained from biopsy of an early stage cervical cancer patient and originally expanded by the Laimins laboratory (39). HFK-E6 and HFK-E7 cell lines were derived by retroviral transduction of HPV31 E6 and E7 into HFKs, followed by selection with G418. All the experiments were conducted with at least three genetically matched HFKs and derived cell lines.

### Organotypic raft cultures

Keratinocytes were seeded onto collagen plugs containing fibroblasts placed on metal grids over growth media, which creates air-liquid interface and grown as organotypic raft cultures for 13 days as described before[61,62].

### Lentiviral silencing

Pre-validated lentiviral shRNAs were purchased from Open Biosystems. Five different shRNAs were individually tested for silencing and pooled shRNAs were used for silencing experiments. Forty-eight hours post transduction; cells were induced to differentiate for further 48 hours in 1.5% methylcellulose. Stably silenced cell lines were made by selecting with puromycin after transduction. Reduction in protein levels was confirmed by western analysis.

### Southern blotting analyses

Total DNA was isolated from cells as described before [8]. DNA was electrophoresed in 0.8% agarose gel and transferred to membranes. Membranes were blocked with salmon sperm DNA containing blocking buffer, and probed with p32-HPV31 probe. After a series of washes with various stringency buffers, membranes were analyzed to autoradiography [63].

### Northern blotting analyses

Total RNA was isolated from cells using STAT60 reagent according to manufacturer’s instructions (Tel-Test Inc.). RNA was electrophoresed in 0.8% agarose gel with formaldehyde and transferred to membranes. Membranes were blocked, and probed with p32-HPV31 probe. After a series of washes with various stringency buffers, membranes were examined by autoradiography [63].

### Immunofluorescence

Cells were grown on treated coverslips with regular E-media for undifferentiated conditions. For differentiation, cells were pretreated with low calcium media for 24hours followed with high calcium for 72 hours. Cells were then fixed with 4% paraformaldehyde, permeabilized with Triton-X100, blocked with normal goat serum and probed overnight for p-KLF4 antibody. After 3 washes with PBS, Alexaflour rabbit secondary antibody was added for one hour and washed thrice in PBS. After a brief incubation with DAPI, coverslips were washed in PBS multiple times before mounting onto glass slides with gelvatol for analysis.

Raft sections were de-paraffinized overnight at 55°C followed by a series of washes in xylene (3), ethanol (3), 70% ethanol and permeabilized with triton x-100. Antigen retrieval was performed in citrate buffer at 95°C for 20 mins. Sections were blocked with normal goat serum (NGS) and probed for 1:200 primary antibodies (KLF4 CST 4038S, pKLF4 Thermo PA5-13081, Loricrin SC-133757, and E1^E4, a kind gift from Sally Roberts) in NGS overnight. After three washes in PBS, Alexaflour secondary antibodies in NGS were added for 1 hour, followed by three washes in PBS. After a brief incubation with DAPI, slides were washed in PBS before mounting coverslips with gelvatol.

### Site directed mutagenesis

Primers for mutations were designed using the Agilent primer design program. Mutagenesis PCRs were performed according to the manufacturer’s instructions (Agilient 200523). The primer sequences are as follows.

URR-R1M-Forward: GTGTTGTGTATGTTGTCCTTATATACACACTATTAGTAACATACTATTACTATTTTA.

Reverse: TAAAATAGTAATAGTATGTTACTAATAGTGTGTATATAAGGACAACATACACAACAC.

URR-R2M-Forward: CCATAGTAAAAGTTGTACACACGGTCCGTTTTTTGCAACTA.

Reverse: TAGTTGCAAAAAACGGACCGTGTGTACAACTTTTACTATGG.

### Chromatin immunoprecipitation assays

ChIP assays were performed as described previously[64]. Primers: KLF4-URR-R1, For: ATGTGTATGTGCTTGTGCTG, Rev: TGACTATTGGGAGGAGCAGG. KLF4-URR-R2, For: ACTTGTTCCTACTTGTTCCTGC, Rev: GCATCAGCATAGTTGTACTAGC.

### Immunoprecipitation assays

Cell lysates were harvested using RIPA buffer with SDS containing protease, phosphatase, and desumoylation inhibitors. Two hundred micrograms of protein sample was immunoprecipitated with protein A/G agarose beads (SC-2003) and KLF4 antibody (SC-393462) overnight. Beads were spun down at 7500 rpm for 2 minutes at 4°C, followed by three cycles of washes with RIPA buffer and spins. Beads were then boiled at 95°C for 5 minutes with Lamelli sample buffer, cooled down, and spun in microcentrifuge. The supernatants were loaded into 8% polyacrylamide gels and western blotting analyses were performed with the following antibodies: Sumo-1 (CST 4930S), and Blimp1 (CST 9115S).

### Holoclone assay

Keratinocytes were seeded sparsely (100–500 cells) with mitomycin treated NIH-3T3 cells in 100 mm dishes and were grown in E-media with twice the amount of Epidermal Growth Factor (10ng/mL) for 3 weeks. The colonies were stained with crystal violet in methanol for 30 minutes, followed by series of water washes.

### RNA sequencing

RNA-sequencing was performed using Illumina HiSeq 2500 NGS platform. Sequencing data were used as input to CRI Illumina RNA-seq pipeline for quality control assessment of raw sequencing data, reads mapping, post-alignment QC, expression quantification, and DEGs identification. The quality of raw sequencing reads was assessed using FastQC v0.11.2, and the post-alignment QC was evaluated with RSeQC v2.3.9[65] and Picard tools v1.117. Reads were mapped to 1). UCSC human genome (hg19) obtained from GATK resource bundle v2.8 using TopHat v2.0.13 [66] guided with UCSC gene annotation model (hg19) obtained from Illumina iGenomes, and 2) HPV31 virus genome with GenBank accession number J04353.1. Gene transcripts were assembled and quantified on human and HPV31 genome separately using Cufflinks v2.2.1 [67] with the hg19 UCSC gene model annotation and HPV31 RefSeq gene annotation as a guide respectively for transcript assembly and bias detection/correction. Sample-based assemblies were merged together using Cuffmerge wrapped in Cufflinks v2.2.1 before quantification of transcripts using Cufflinks wrapped method Cuffnorm and count-based method featureCounts[68]. DEGs were identified between 4 various group comparisons using Cuffdiff wrapped in Cufflinks v2.2.1. During the entire analysis, R (R Core Team, 2014) were used to assist in the exploration and summarization of the analysis results.

### Plasmids

KLF4 expression vector was purchased from Addgene. Blimp1 expression vector was a kind gift from Shannon Kenney. pBR322-HPV31gen, pUC-HPV16gen, URR-pro, pLxSn 31-E6, pLxSn 31-E7, and Lpro-luc were described before [18,69,70].

## Supporting Information

S1 Table Global targets of KLF4. KLF4-silenced HFKs and HFK-31gen cells were differentiated in methylcellulose, mRNAs were isolated and RNA-seq analyses were performed. A threshold criteria of at least 1.5-fold change following KLF4 knock down, and a minimum of 100 reads were applied to eliminate low expressing genes. Targets of KLF4 are presented in a Microsoft Excel table as a ratio of KLF4-silenced cells over control (shGFP) in differentiated conditions of HFKs and HFK-31gen cells. Ratio value ≥ 1.5 represents activation and ratio value ≤ 0.66 represents repression.
(XLSX)

S1 Fig KLF4 is required for HPV DNA amplification. KLF4 was transiently silenced in CIN-612 cells by individually infecting with the three different lentiviral shRNAs that target different regions of the KLF4 gene. Differentiation was induced by suspending cells in methylcellulose. The reductions in KLF4 protein levels were observed by western analysis in both undifferentiated and differentiated conditions of shKLF4 cells compared to mock and shGFP controls. Silencing KLF4 with shRNAs impaired the ability of the cells to amplify episomal DNA upon differentiation as shown by Southern blot analysis.
(TIF)

S2 Fig KLF4 binding to the viral URR is specific. KLF4 and IgG immunoprecipitated DNA were analyzed for enrichment of GAPDH genomic sequences and 18srDNA. KLF4 did not display enriched binding to either region compared to IgG controls, emphasizing KLF4 binding to the viral URR is specific.
(TIF)

S3 Fig Expression of KLF4 target genes in HFKs and HPV-positive cells. After determining the targets of KLF4 using KLF4-depleted cells in RNA-seq, the levels of the targets were analyzed using control-differentiated samples (shGFP) from HFKs and HFK-31gen cells. The results are represented as fold-increase/decrease in HFK-31gen over HFK samples. A subset of differentiation-associated factors was increased in HFK-31gen cells as compared to HFKs and a subset of cell adhesion-associated markers was repressed in HFK-31gen cells over HFKs.
(TIF)

S4 Fig Heat maps of differentially regulated KLF4 targets. KLF4 targets that were differentially regulated in HFKs and HFK-31gen cells upon silencing of KLF4 during differentiation are represented as heat maps. The targets are categorized according to their known cellular functions.
(TIF)

S5 Fig KLF4 targets that were oppositely regulated in HFKs and HPV-positive cells. A list of KLF4 target genes that were suppressed in HFKs but activated in HFK-31gen cells upon KLF4 silencing.
(TIF)

S6 Fig KLF4 requirement in HPV-16 keratinocytes mirrors HPV-31. (S6A Fig). KLF4 was stably silenced in HPV-16gen keratinocytes with lentiviruses expressing shRNAs. KLF4 protein levels were reduced in shKLF4 cells compared to controls as shown in the western blot. (S6B Fig). KLF4 silenced HFK-16gen cells formed rafts similar to HFK-31gen cells with morphologically altered cornified envelope Immunostaining experiments showed reduction in KLF4 staining specifically in shKLF4 rafts compared to controls. Loricrin staining was absent in shKLF4 rafts compared to controls. (S6C Fig). Southern blot showing the maintenance of HPV16 genomes as episomes and their amplification upon differentiation.
(TIF)

S7 Fig NFκB activity in HPV-31 keratinocytes. (S7A Fig). NFκB activity was measured using NFκB-reporter construct and was found to be suppressed in HPV31 keratinocytes compared to HFKs. (S7B Fig). The active subunit of NFκB pathway, p65, activated miR-145 promoter in a dose-dependent manner.
(TIF)
