# Supplementary figures and images for: Post-Transcriptional Regulation of KLF4 by High-Risk Human Papillomaviruses Is Necessary for the Differentiation-Dependent Viral Life Cycle
Source: PLoS Pathog. 2016 Jul 7;12(7):e1005747. doi: 10.1371/journal.ppat.1005747 (PMC4936677; doi:10.1371/journal.ppat.1005747)

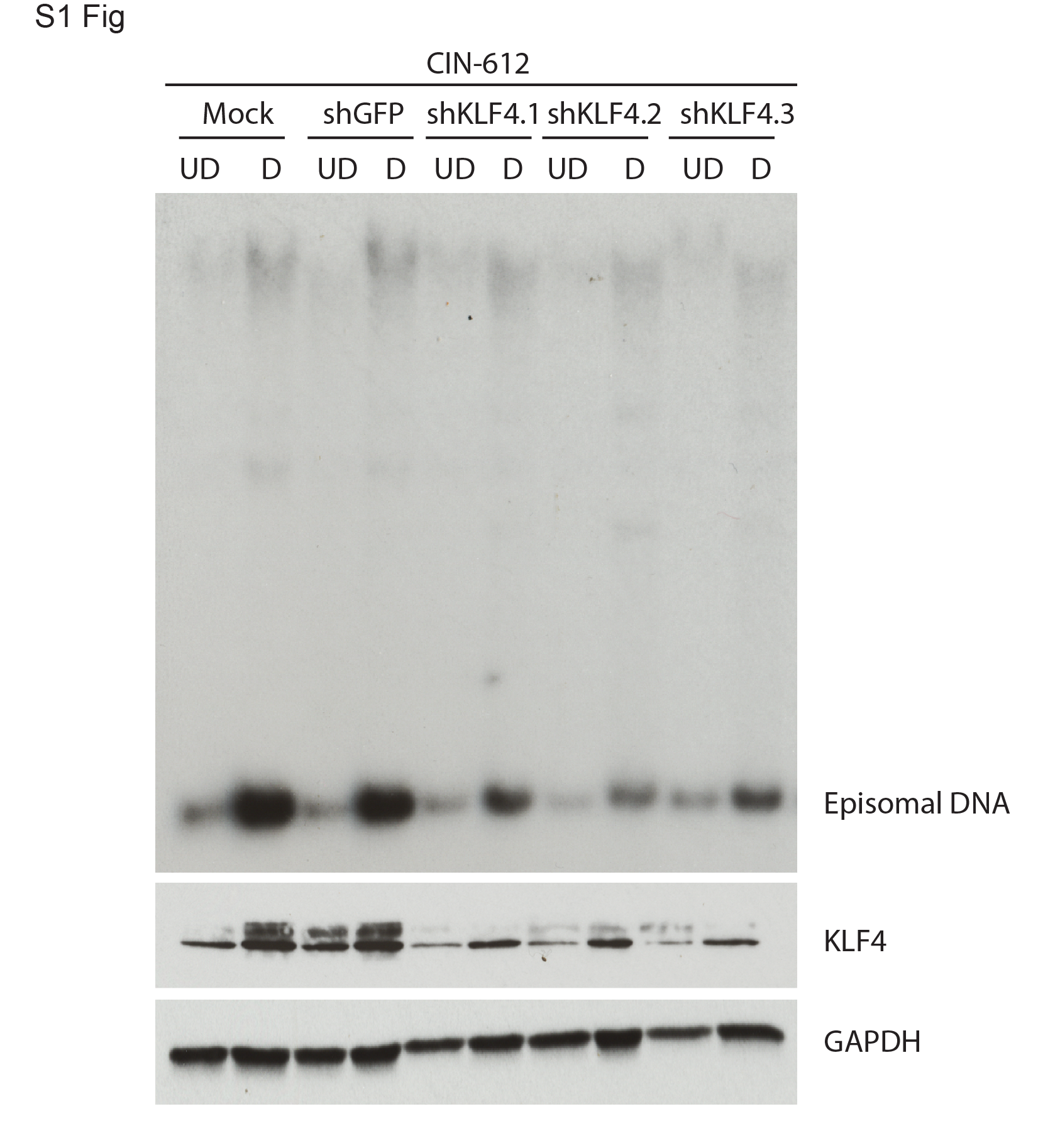

Supplement: S1 Fig — KLF4 was transiently silenced in CIN-612 cells by individually infecting with the three different lentiviral shRNAs that target different regions of the KLF4 gene. Differentiation was induced by suspending cells in methylcellulose. The reductions in KLF4 protein levels were observed by western analysis in both undifferentiated and differentiated conditions of shKLF4 cells compared to mock and shGFP controls. Silencing KLF4 with shRNAs impaired the ability of the cells to amplify episomal DNA upon differentiation as shown by Southern blot analysis. (TIF) [file ppat.1005747.s002.tif]

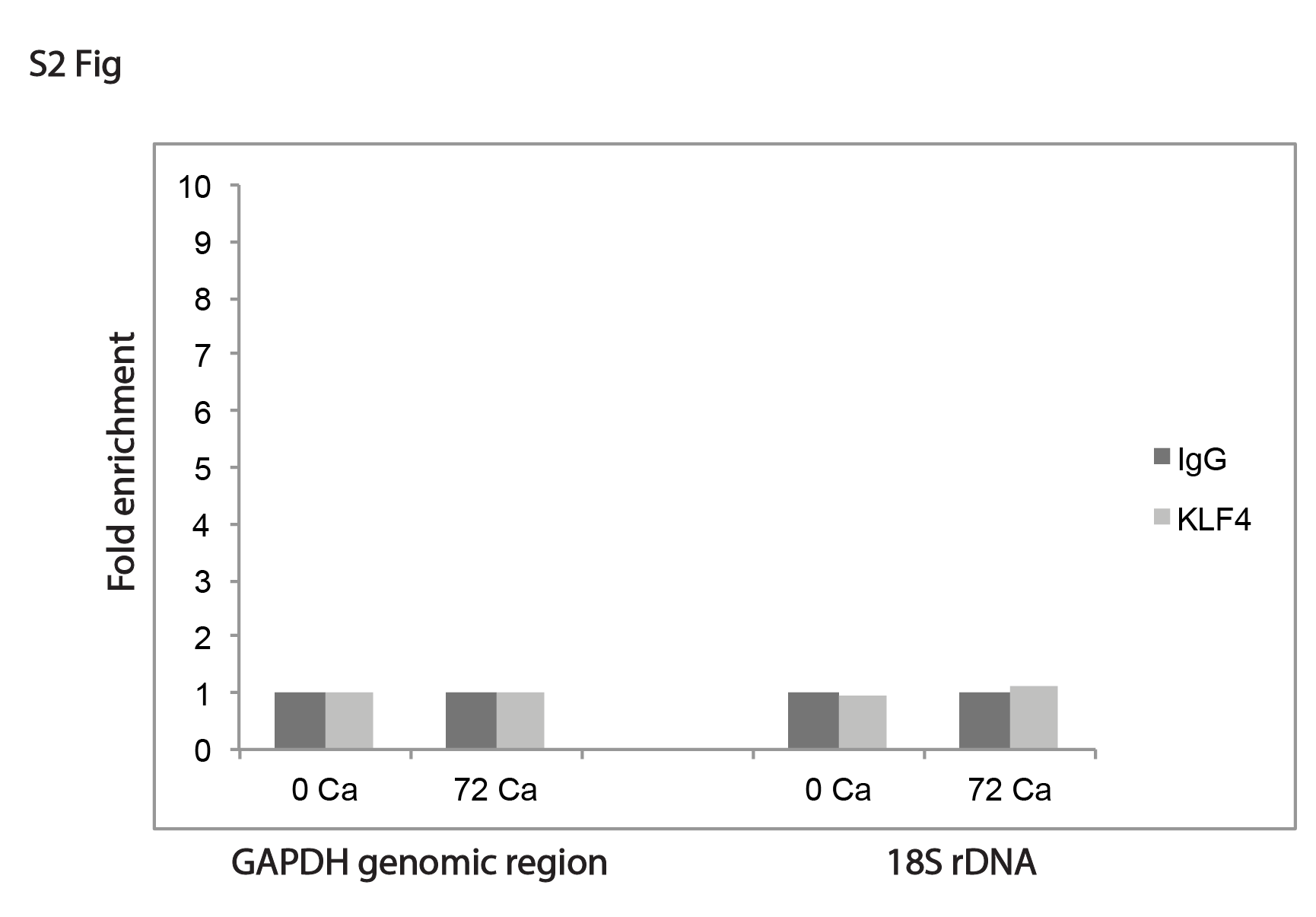

Supplement: S2 Fig — KLF4 and IgG immunoprecipitated DNA were analyzed for enrichment of GAPDH genomic sequences and 18srDNA. KLF4 did not display enriched binding to either region compared to IgG controls, emphasizing KLF4 binding to the viral URR is specific. (TIF) [file ppat.1005747.s003.tif]

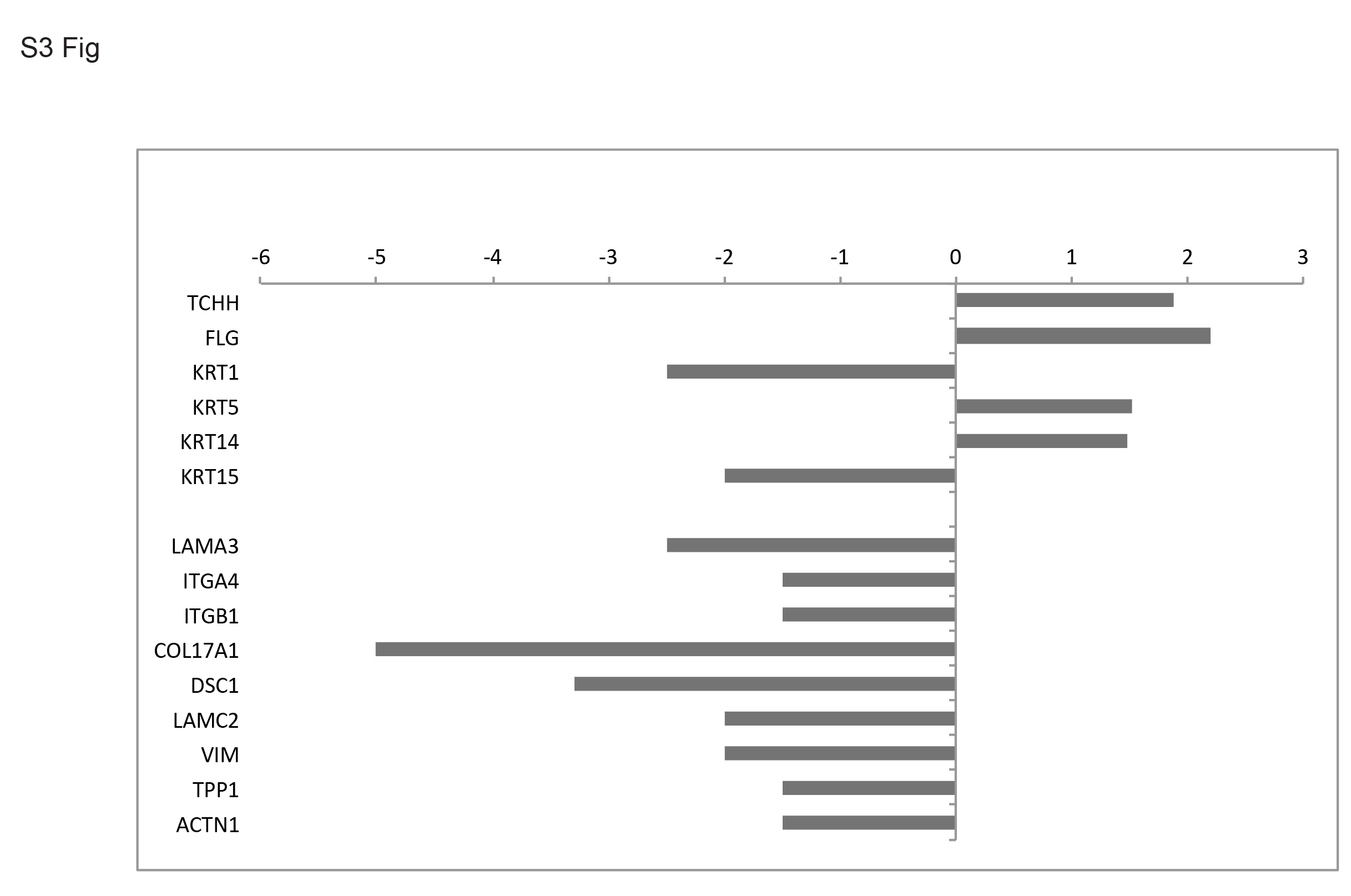

Supplement: S3 Fig — After determining the targets of KLF4 using KLF4-depleted cells in RNA-seq, the levels of the targets were analyzed using control-differentiated samples (shGFP) from HFKs and HFK-31gen cells. The results are represented as fold-increase/decrease in HFK-31gen over HFK samples. A subset of differentiation-associated factors was increased in HFK-31gen cells as compared to HFKs and a subset of cell adhesion-associated markers was repressed in HFK-31gen cells over HFKs. (TIF) [file ppat.1005747.s004.tif]

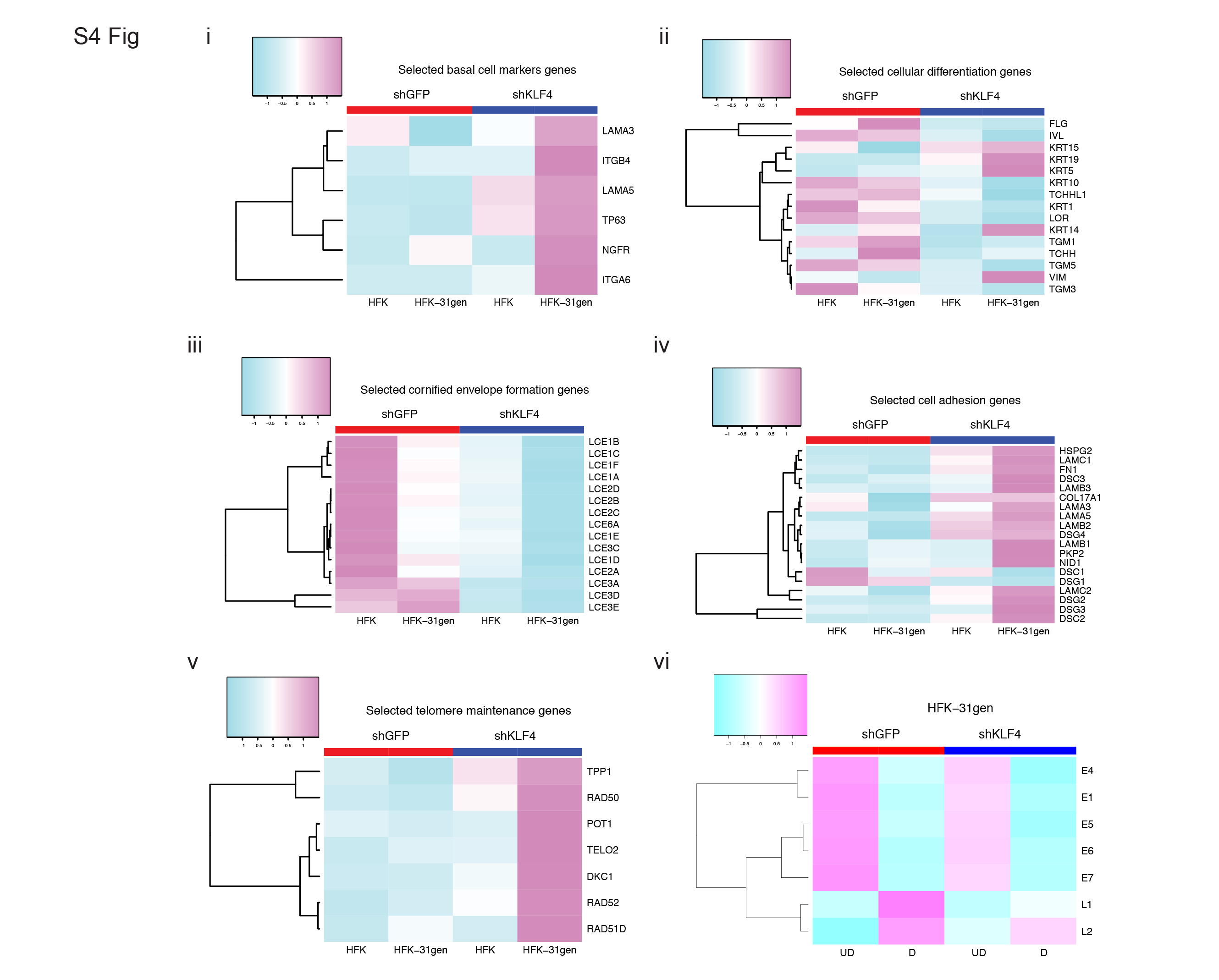

Supplement: S4 Fig — KLF4 targets that were differentially regulated in HFKs and HFK-31gen cells upon silencing of KLF4 during differentiation are represented as heat maps. The targets are categorized according to their known cellular functions. (TIF) [file ppat.1005747.s005.tif]

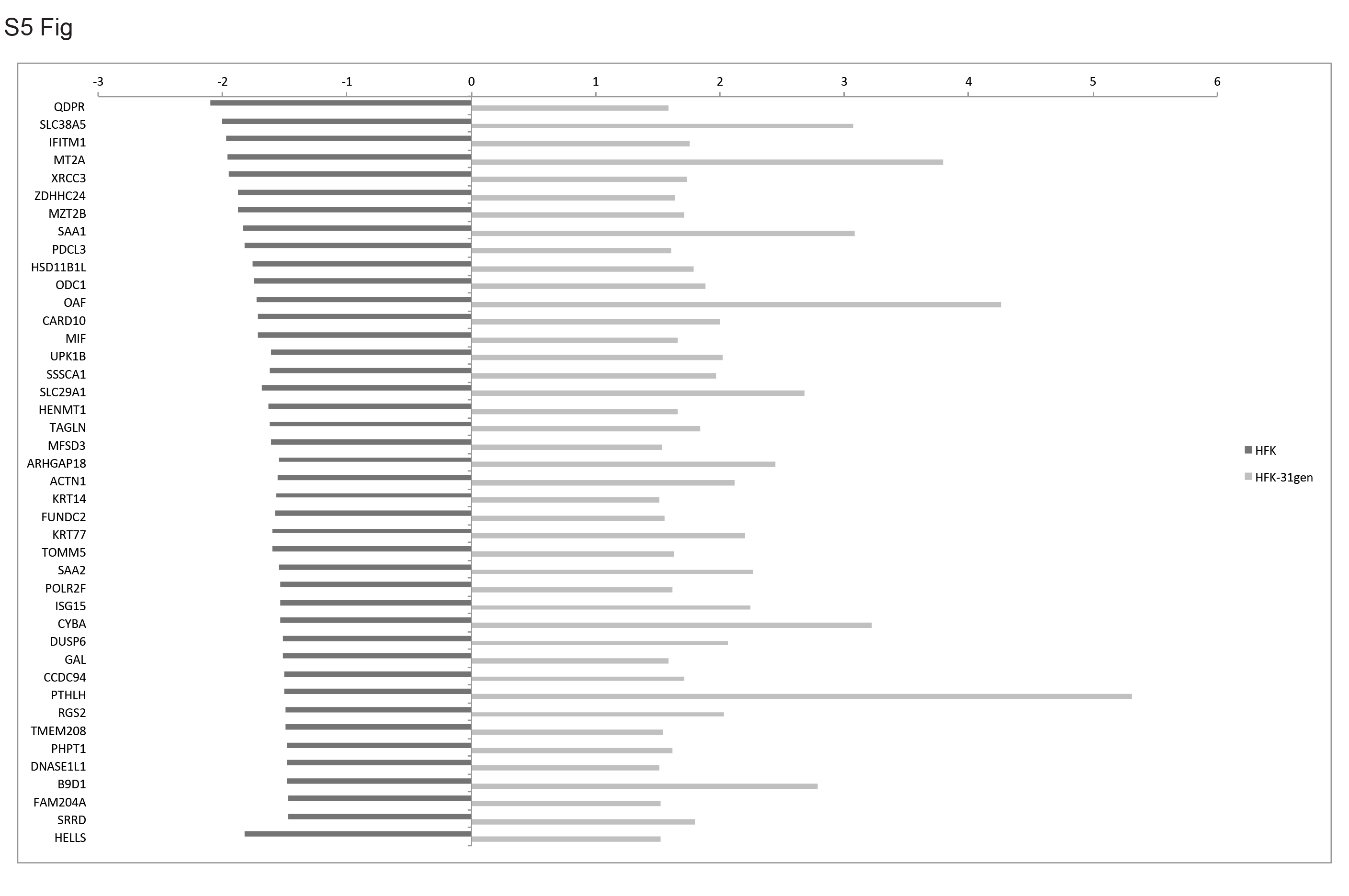

Supplement: S5 Fig — A list of KLF4 target genes that were suppressed in HFKs but activated in HFK-31gen cells upon KLF4 silencing. (TIF) [file ppat.1005747.s006.tif]

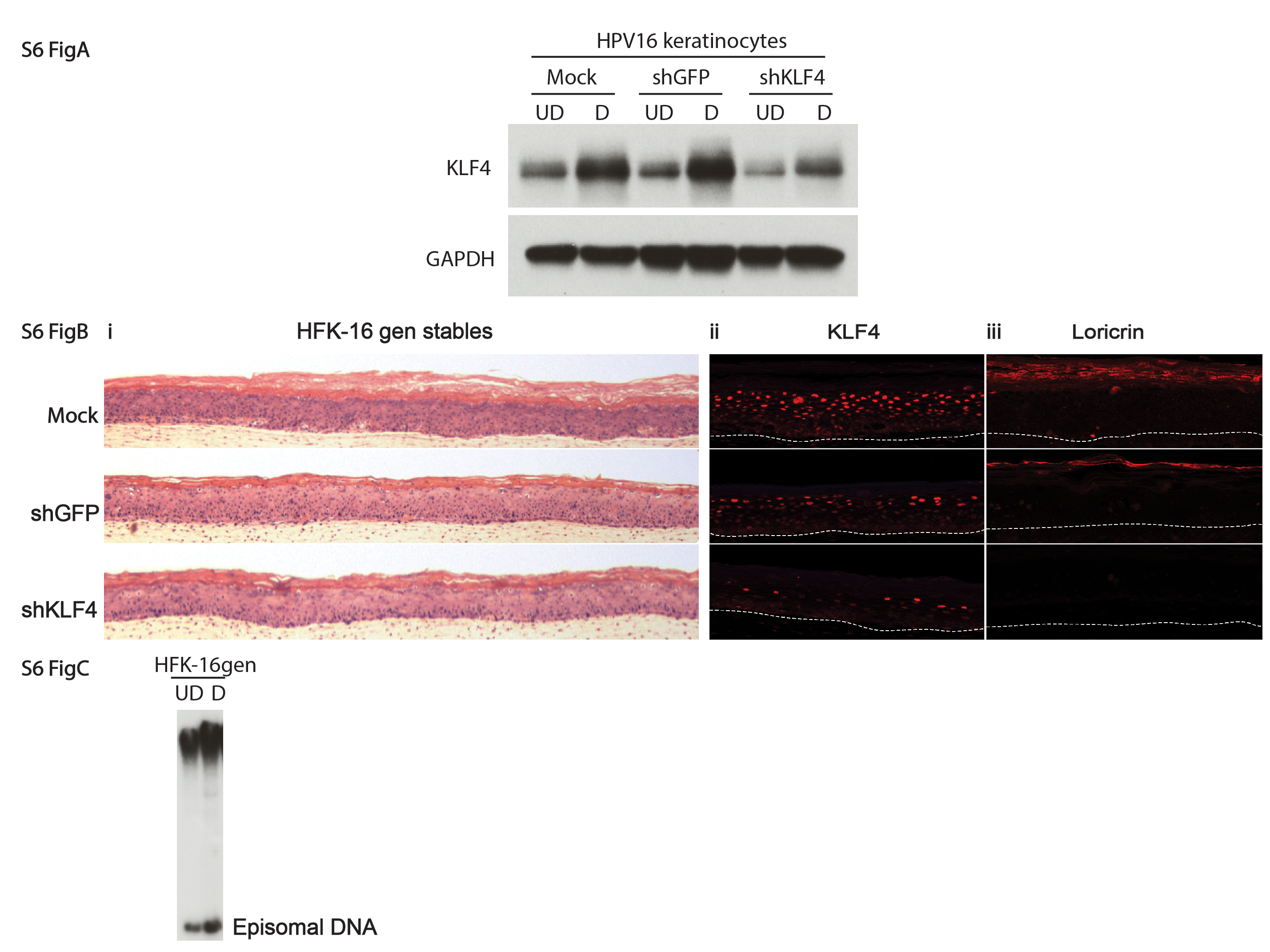

Supplement: S6 Fig — (S6A Fig). KLF4 was stably silenced in HPV-16gen keratinocytes with lentiviruses expressing shRNAs. KLF4 protein levels were reduced in shKLF4 cells compared to controls as shown in the western blot. (S6B Fig). KLF4 silenced HFK-16gen cells formed rafts similar to HFK-31gen cells with morphologically altered cornified envelope Immunostaining experiments showed reduction in KLF4 staining specifically in shKLF4 rafts compared to controls. Loricrin staining was absent in shKLF4 rafts compared to controls. (S6C Fig). Southern blot showing the maintenance of HPV16 genomes as episomes and their amplification upon differentiation. (TIF) [file ppat.1005747.s007.tif]

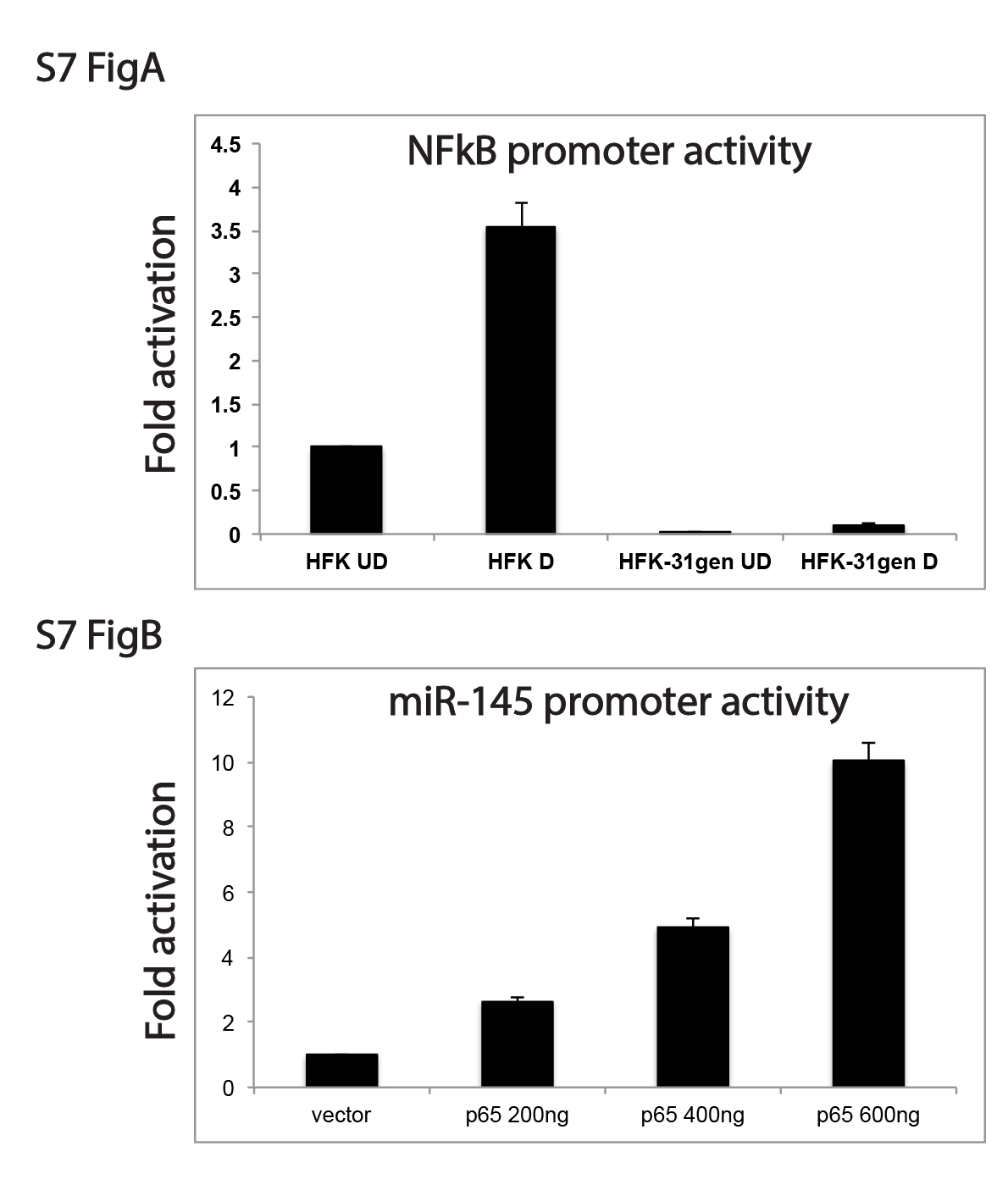

Supplement: S7 Fig — (S7A Fig). NFκB activity was measured using NFκB-reporter construct and was found to be suppressed in HPV31 keratinocytes compared to HFKs. (S7B Fig). The active subunit of NFκB pathway, p65, activated miR-145 promoter in a dose-dependent manner. (TIF) [file ppat.1005747.s008.tif]
